# The thymus gland is a target and maintainer of amyotrophic lateral sclerosis pathophysiology

**DOI:** 10.1016/j.bbih.2026.101323

**Published:** 2026-09-06

**Authors:** Julia Pereira Lemos, Daniel Stockholm, Brenda Dinatale, Amanda Silva Chaves, Sonia Pezet, Vinicius Frias Carvalho, Ana Rosa Pérez, Daniella Arêas Mendes-da-Cruz, Wilson Savino, Piera Smeriglio

**Affiliations:** a Sorbonne Université, INSERM, Institut de Myologie, Centre de Recherche en Myologie, Paris, F-75013, France; b PSL Research University, EPHE, Paris, F-75014, France; c Sorbonne Université, INSERM, Centre de Recherche Saint-Antoine, Paris, F-75012, France; d Institute of Clinical and Experimental Immunology of Rosario (IDICER-CONICET), Faculty of Medical Sciences, National University of Rosario, Rosario, Argentina; e Laboratory of Inflammation, Oswaldo Cruz Institute, Oswaldo Cruz Foundation, Rio de Janeiro, Brazil; f National Institute of Science and Technology on Neuroimmunomodulation (INCT-NIM), Oswaldo Cruz Institute, Oswaldo Cruz Foundation, Rio de Janeiro, Brazil; g Rio de Janeiro Research Network on Neuroinflammation (RENEURIN), Oswaldo Cruz Institute, Oswaldo Cruz Foundation, Rio de Janeiro, Brazil; h INOVA-IOC Network on Neuroimmunomodulation, Oswaldo Cruz Institute, Oswaldo Cruz Foundation, Rio de Janeiro, Brazil; i Center for Research and Production of Biological Reagents (CIPReB), Faculty of Medical Sciences, National University of Rosario, Rosario, Argentina; j Laboratory on Thymus Research, Oswaldo Cruz Institute, Oswaldo Cruz Foundation, Rio de Janeiro, Brazil

**Keywords:** Amyotrophic lateral sclerosis (ALS), SOD1-G93A mouse model, Thymus, Regulatory T cells (Treg), Thymocyte development, Immune homeostasis

## Abstract

Amyotrophic lateral sclerosis (ALS) is a fatal neurodegenerative disease characterized by the selective loss of motor neurons in the brain and spinal cord with evidence of local neuroinflammation. Regulatory T cells (Treg) exert neuroprotective effects and correlate with disease progression, but central mechanisms governing Treg development during ALS remain unclear. We investigated the thymic architecture and T cell differentiation in SOD1-G93A (mSOD1) ALS mice at multiple disease stages. Thymocyte and thymic epithelial cells (TEC) subpopulations, as well as stages of Treg differentiation and suppressive capacity, were analyzed by flow cytometry, *in situ* immunofluorescence, and functional assays. At late disease stage (120 days-old), mSOD1 mice displayed thymic atrophy, reduced thymocyte numbers, and decreased double-positive (DP), CD4 single-positive (SP), and Treg populations. Foxp3 expression per cell was preserved, but Treg progenitors and mature Treg numbers declined, paralleled by reduced suppressive function *in vitro*. TEC analysis revealed reduced total and mature medullary TEC (CD80^high^MHCII^high^), despite preserved Aire expression. Foxp3^+^ cells were abnormally localized at cortical sites, away from mTEC, associated with migratory abnormalities and increased apoptosis. TREC evaluation revealed reduced intrathymic sjTREC, consistent with impaired proliferation and differentiation between DN and DP stages, although peripheral sj/βTREC ratios remained stable. Reduced Treg numbers were also observed in lymph nodes draining affected hindlimbs. Our findings identify the thymus as a target organ in ALS, where thymic involution, TEC loss, and Treg dysfunction may compromise immune tolerance. These alterations likely reinforce the role of the thymus in peripheral immune dysregulation and neuroinflammation observed in ALS.

## Highlights

• Thymus shrinks in late ALS, reducing total thymocytes and CD4 cells.
• Regulatory T cells decline in thymus and periphery of ALS mice.
• Medullary thymic epithelial cells are reduced in the thymus of late-stage ALS mice.
• Thymic defects reflect peripheral immune imbalance, worsening ALS progression.

## 1 Introduction

Amyotrophic lateral sclerosis (ALS) is a progressive neurodegenerative disorder characterized by the selective loss of motor neurons, leading to skeletal muscle paralysis (Valko and Ciesla, 2019). ALS primarily affects middle-aged adults (Iyer et al., 2018) and is the most prevalent motor neuron disease in this population (Bensimon et al., 1994). Patients experience muscle weakness due to ongoing atrophy, which ultimately progresses to complete paralysis and, in most cases, death from respiratory failure within three to five years following diagnosis (Iyer and Jones, 2017; Ravits and La Spada, 2009). Currently, there is no cure, and existing treatments are mainly aimed at managing symptoms. Approximately 10% of ALS cases are familial (fALS), resulting from genetic mutations that are most often inherited in an autosomal dominant pattern (Rajabinejad et al., 2020). Among these, around 20% are linked to mutations in the Cu/Zn superoxide dismutase 1 (mSOD1) gene, and 40% are associated with mutations in the chromosome 9 open reading frame 72 (C9orf72) gene, particularly in European and North American populations, though the prevalence of these mutations varies significantly in Asian cohorts (Nardo et al., 2016; Rajabinejad et al., 2020; Suzuki et al., 2023).

In both sporadic and familial forms of ALS, disease progression is characterized by the selective degeneration of motor neurons within the spinal cord, frequently accompanied by localized neuroinflammatory responses. A hallmark of this pathological process is the activation of glial cells, namely microglia and astrocytes, which actively contribute to disease progression by mediating neuroinflammation (Bowerman et al., 2018). Although neuroinflammation is not considered the primary trigger of ALS, it is strongly associated with disease exacerbation. The interplay between activated microglia, astrocytes, and the adaptive immune system is underscored by the crosstalk between central nervous system-resident glial cells and infiltrating peripheral immune cells, including T lymphocytes. Notably, post-mortem analyses of ALS patients revealed the presence of reactive microglia in conjunction with infiltrating T cells (Kawamata et al., 1992). While the precise alterations in T cell populations in ALS remain a matter of debate, several studies have reported a reduction in circulating CD4^+^ T cells (Chen et al., 2014) and an increased prevalence of circulant proinflammatory effector T cell subsets in affected individuals (Saresella et al., 2013; Shi et al., 2007).

While some aspects of the CD4^+^ T cell response are detrimental during ALS progression, research has shown that complete deletion of CD4^+^ T cells actually accelerates the disease. In experiments where mSOD1 mice were crossed with T cell receptor beta chain (TCRβ) deficient mice (TCRβ^−/−^), which lack TCRαβ^+^CD4^+^ and TCRαβ^+^CD8^+^ T cells, there was an accelerated disease progression, although the onset of the disease remained unchanged (Chiu et al., 2008). Similar findings were observed when mSOD1 mice were crossed with Rag2^−/−^ mice (which lack both B and T lymphocytes) or CD4^−/−^ mice (deficient in CD4^+^ T lymphocytes) (Beers et al., 2008). Collectively, these studies highlight the neuroprotective function of CD4^+^ T cells, particularly CD4^+^CD25^+^ and CD4^+^CD25^+^Foxp3^+^ regulatory T (Treg) cells and suggest that the phenotypes of T cells at diagnosis could serve as reliable predictors for disease prognosis (Yazdani et al., 2022).

The infiltration of CD4^+^CD25^+^Foxp3^+^ Treg cells into the central nervous system has been shown to attenuate neuroinflammation and promote the activation of neuroprotective microglia in murine models of ALS (Sheean et al., 2018). This immunomodulatory effect is associated with elevated levels of anti-inflammatory cytokines and a shift toward a neuroprotective glial phenotype, particularly during the slower phase of disease progression (Beers et al., 2011). As ALS advances, however, pro-inflammatory cytokine levels increase, coinciding with a decline in Treg populations, potentially due to the downregulation or loss of Foxp3 expression (Beers et al., 2011). Parallel findings have been reported in ALS patients, where Treg levels are initially elevated during the early, more indolent phase of the disease but subsequently decrease as disease progression accelerates, likely due to impaired FOXP3 expression and Treg development (Beers et al., 2011; Henkel et al., 2013). Furthermore, Treg isolated from patients exhibiting rapid disease progression demonstrate a diminished capacity to suppress the proliferation of autologous effector T cells compared to Treg from slow-progressing patients or healthy individuals (Beers et al., 2017). This impaired suppressive function correlates with reduced FOXP3 mRNA and protein levels, highlighting the essential role of stable FOXP3 expression in preserving Treg-mediated immunoregulatory activity (Beers et al., 2017).

A key remaining question is whether or not a defect of Treg in ALS could be seen in early differentiation stages of intrathymic development. The thymic production of central Treg and their migration to peripheral immune compartments are critical for maintaining self-tolerance and preventing autoimmunity; consequently, deficiencies in central Treg have been linked to autoimmune pathologies (Wing et al., 2019). In boxes 1 and 2 seen below, we summarize basic knowledge related to intrathymic T cell development and the interaction with non-lymphoid thymic microenvironmental cells.

Box 1. Thymic development of conventional and regulatory T cells. Central Treg are generated within the thymus, a primary lymphoid organ essential for the maturation and differentiation of T lymphocytes (Takaba and Takayanagi, 2017). This developmental process is orchestrated through interactions between developing thymocytes and the thymic microenvironment, particularly thymic epithelial cells (TEC). Based on the CD4 and CD8 co-receptor expression, four major populations can be defined along with the order of thymocyte differentiation: double-negative (DN), are the most immature cells, and do not express the co-receptors (CD4^−^CD8^−^). DN thymocytes can be further subdivided, based on the expression of CD44 and CD25 molecules, into four phenotypic stages called DN1 (CD44^+^CD25^−^), DN2 (CD44^+^CD25^+^), DN3 (CD44^−^CD25^+^) and DN4 (CD44^−^CD25^−^). Double-positive thymocytes (DP), which express both CD4 and CD8 (CD4^+^CD8^+^), account for around 80% of thymocytes. CD4 and CD8 single-positive (SP) cells (CD4^+^CD8^−^ and CD4^−^CD8^+^, respectively), correspond to the final stage of development, where T cells are ready to leave the thymus and exercise their effector functions in the periphery, after passing through the positive and negative selection processes within the thymus, which prevent the development of T cells with autoimmune potential (Ruiz Pérez et al., 2024). Interestingly, developing thymocytes that recognize, with a certain avidity, the antigens presented by the class II MHC of mTEC can undergo a clonal shift, comprising a series of stimuli that lead to the generation of CD4^+^CD25^+^Foxp3^+^ thymic regulatory T cells (Treg) (Josefowicz et al., 2012; Ohkura and Sakaguchi, 2010).

Box 2. Thymic microenvironment and cell migration during T cell differentiation. In addition to the cellular components of the thymus, during the differentiation process, thymocytes encounter different components of the thymic microenvironment, such as cortical and medullary TEC. This three-dimensional cellular organization is interspersed with a network of extracellular matrix (ECM) that structures and supports the organ. The maturation occurs simultaneously with migration, so that more immature DN and DP cells are found in the cortex, while mature SP cells are found in the medulla. Migration is mediated by various molecular interactions, such as between integrins and ECM proteins and chemokines and chemokine receptors. ECM proteins form a bridge between thymocytes and the cells of the thymic microenvironment, both expressing their receptors. This interaction is crucial, since cell migration is the result of the adhesion and de-adhesion of thymocytes to the substrate, while chemokines produced by the thymic microenvironment direct the migration and differentiation of these cells (Savino et al., 2002, 2004).

Although research specifically addressing a putative thymic involvement in ALS remains limited, available data suggests a thymic dysfunction in ALS patients harboring SOD1 mutations as well as in the mSOD1 mouse model (Seksenyan et al., 2010). In the present study, we provide novel evidence demonstrating that, as ALS progresses, the thymus is directly affected by the disease, resulting in thymic atrophy, reduced thymocyte intrathymic proliferation and reduction in the Treg number, which is mirrored in peripheral lymphoid tissues. These findings suggest that the thymus itself is a target organ in ALS, both reflecting and potentially reinforcing the peripheral immune phenotype, characterized by diminished Treg cells and subsequent exacerbation of motor neuron degeneration.

## 2 Materials and methods

### 2.1 Mouse maintenance and husbandry

B6SJL-Tg(SOD1*G93A)1Gur/J (mSOD1; common name: SOD1-G93A) and B6SJLF1/J (WT) were purchased from The Jackson Laboratory. Mice were bred and maintained under controlled conditions following the French and European guidelines for the use of animal models (2010/63/EU). All the mice were bred in the Sorbonne University/INSERM animal facility in a temperature/humidity-controlled room (22 °C/55% humidity), under a 12 h light/dark cycle, specific pathogen-free conditions, and with access to food and water *ad libitum*. Genotyping of mSOD1 mice was carried out by PCR and human SOD1 genome copy number assessed as described by Jackson Laboratory. The mSOD1 offspring aged 30, 60, 90 and 120 days and littermate WT, as controls, were used in the different experiments described below. All protocols for the use and care of animals were approved by the French Ministry of Higher Education and Research, and humane endpoints were established in the animal protocol (Agreement number #31040 2021041418286649 v4).

### 2.2 Thymus relative weight

Control and mSOD1 mice were weighed. Following euthanasia with deep anesthesia using xylazine (20 mg/kg i.p.) and ketamine (200 mg/kg i.p.), thymuses were collected, cleaned and weighed. The relative organ weight for each animal was calculated using the following equation: thymus/mouse weight (mg/g).

### 2.3 Flow cytometry

#### 2.3.1 Lymphocyte staining

For lymphocyte staining, single-cell suspensions from thymi, inguinal lymph nodes, axillary lymph nodes and spleens were prepared by mechanical disruption of the tissues through a 70-μm mesh in phosphate-buffered saline (PBS) (Gibco™) and manually counted using a KOVA™ Glasstic™ Slide 10 with Grids (Kova International Inc.). After counting, one million cells were separated for staining with viability dye for 20 min at room temperature, followed by surface antibody stains for 30 min at 4 °C, then washed with 2% FBS (Gibco™) in PBS and fixed using the eBioscience™ FOXP3/Transcription Factor Staining Buffer Set (Invitrogen™). For intracellular staining, cells were incubated for 30 min at 4 °C with the appropriate antibodies.

#### 2.3.2 Thymic epithelial cell staining

For microenvironmental cell staining, single-cell suspensions from thymi were prepared by enzymatic digestion with 0.25 mg/ml papain (Worthington Biochemical Corporation), 0.25 mg/ml collagenase IV (Gibco™) and 0.1 mg/ml DNase I (Roche) in DMEM/F12 (Dulbecco's Modified Eagle Medium/Nutrient Mixture F-12; Gibco™). Digestion was followed by EpCAM^+^ cells enrichment through positive magnetic selection using LS columns and Quadro MACS (Miltenyi Biotec). Briefly, cells were stained with EpCAM-APC/Cy7 antibody at 4 °C for 15 min and, after washing, anti-Cy7 MicroBeads (Miltenyi Biotec; Catalog number 130-091-652) were added for 15 min at 4 °C. After washing with a buffer containing 0.5% bovine serum albumin (BSA) (Sigma Aldrich)/2 mM EDTA (Invitrogen™) diluted in PBS (Gibco™), magnetic separation was performed according to Miltenyi's protocol. After cell suspension, the totality of cells was stained with viability dye for 20 min at room temperature, followed by surface antibody stains for 30 min at 4 °C, then washed with 2% FBS (Gibco™) in PBS.

#### 2.3.3 Cell death assay staining

For cell death assays, one million lymphocytes were stained with viability dye for 20 min at room temperature. After washing with PBS (Gibco™), cells were incubated with FITC Annexin V diluted in Annexin V Binding Buffer (Biolegend®) for 15 min at room temperature and analyzed immediately.

All samples were acquired on CytoFLEX (Beckman Coulter Inc.) using CytExpert Software and the analysis was done using FlowJo v.10.8.1 software (Tree Star). All antibodies used in flow cytometry are listed in Supplementary Table 1.

### 2.4 Western blotting

Freshly frozen thymi were put in Lysing Matrix D, 2 mL tubes (MP Biomedicals), in a lysis buffer containing 1X RIPA (Thermofisher), 1X cOmplete™, Mini, EDTA-free Protease Inhibitor Cocktail (Roche) and 0.1% SDS (Invitrogen) and disrupted in a Benchmark BeadBug™ 6 Homogenizer (FastPrep FP120) (Sigma-Aldrich) for protein extraction. Protein extracts were quantified using DC™ Protein Assay Kit I (BioRad; Catalog number 5000111). Proteins were resolved on a 12% polyacrylamide precast gel (Criterion XT Bis-Tris Gel, 12%, 12+2-well; BioRad Catalog number 3450117) and transferred with Trans-Blot Turbo Transfer System (BioRad; Catalog number 1704150) to nitrocellulose membranes (TransBlot® TurboTM Midi-size nitrocellulose; BioRad; Catalog number 1704271). Membranes were blocked with 5% skim milk/1X Tris-Buffered Saline, 0.1% Tween® 20 Detergent (TBST) for 1 h at room temperature (RT). The membranes were then labeled overnight at 4 °C, under agitation, with the primary antibodies diluted in 5% skim milk/TBST. The day after, the membranes were washed with 5% skim milk/TBST, followed by incubation during 45 minutes-1 hour at RT, under agitation, with Horseradish peroxidase (HRP)-conjugated secondary antibody (Supplementary Table 1) diluted in TBST/0.5% skim milk. After washing, the signal was developed using the SuperSignal West Dura Kit (Thermofisher; Catalog number 34076), according to manufacturer's user guide (Document version MAN0011307 Rev. B.0). Images were acquired in a ChemiDoc MP Imaging System (BioRad) and quantified using the Image J software (Rasband, WS ImageJ, NIH). Antibodies used in immunoblotting are listed in Supplementary Table 1.

### *2.5* *In situ* hybridization

Thymi were removed from mSOD1 and WT mice, embedded in Tissue-Tek O.C.T. Compound (Sakura Finetechnical Co.) and maintained at −80 °C. Ten μm thick cryostat sections were settled on SuperFrost® Plus, Menzel Gläser slides (Thermo Scientific). Fresh-frozen sample preparation, pretreatment and RNAscope assay were performed using the RNAscope™ Multiplex Fluorescent Reagent Kit v2 (ACD Bio), according to the user manual (Document number 323100). For human superoxide dismutase 1 (SOD1) mRNA specific hybridization, the probe RNAscope™ Probe - Hs-SOD1-No-XMm-C1 (ACD Bio; Catalog Number 1147991-C1) was used and detection was possible using the Opal™ 570 dye (Akoya Biosciences; Catalog Number FP1488001KT). At the end of the protocol, slides were mounted using ProLong™ Gold Antifade mounting medium (Thermo Fisher Scientific), the images were obtained as tiles with 3% overlap using a Plan Apochromatic λ 20× objective with a numerical aperture of 0.75 on a Nikon Ti2 Spinning Disk Confocal microscope equipped with a Color Camera Nikon DS-Ri2 (Nikon Instruments Inc.).

### *2.6* *In situ* immunofluorescence

Thymi were removed from mSOD1 and WT mice, embedded in Tissue-Tek Optimal Cutting Temperature Compound (Sakura Finetechnical Co.) and maintained at −80 °C. Four 5-μm-thick cryostat sections at a distance of 200 μm between them – in order to achieve different representative regions of the organ – were obtained for each organ, settled on SuperFrost® Plus, Menzel Gläser slides (Thermo Scientific) and acetone-fixed for 10 min at −20 °C. Slides were treated with PBS 1% BSA for 30 min at room temperature and incubated with primary antibodies (Supplementary Table 1) diluted in PBS/1% BSA/0.1% Saponin (Sigma Aldrich) overnight at 4 °C. Samples were then submitted to corresponding secondary antibodies for 30 min at room temperature when necessary. Nucleic acid staining was performed with DAPI (Sigma Aldrich) for 5 min at room temperature. After mounting the slides using ProLong™ Gold Antifade mounting medium (Thermo Fisher Scientific), the images were obtained with a Plan Apochromatic λ 20× objective with a numerical aperture of 0.75 using the Nikon Ti2 Spinning Disk Confocal microscope equipped with a Color Camera Nikon DS-Ri2 (Nikon Instruments Inc.). Sections were acquired as tiles with 3% overlap. Immunostained thymus sections were analyzed using QuPath software (version 0.5.1; (Bankhead et al., 2017). All antibodies used in *in situ* immunofluorescence experiments are listed in Supplementary Table 1, and details of the QuPath-based image analyses are provided in supplementary Method 1.

### 2.7 Corticosterone quantification

Following euthanasia with deep anesthesia using xylazine (20 mg/kg i.p.) and ketamine (200 mg/kg i.p.), blood samples were promptly collected in microcentrifuge tubes. Blood was allowed to clot by leaving it undisturbed at room temperature for 15-30 min. The samples were then centrifuged at 5000 x g for 10 min and stored at −80 °C until analysis. Corticosterone levels were measured using the Corticosterone ELISA Kit (Cayman Chemical; Catalog number 501320) in accordance with the manufacturer's instructions.

### *2.8* *In vitro* differentiation assay

Thymi from mSOD1 and WT mice were prepared by mechanical disruption of the tissues through a 70-μm mesh in 2% FBS in PBS. After counting as described above, CD4 negative selection using Mouse CD4^+^ T Cell Isolation Kit (Miltenyi Biotec; Catalog number 130-104-454) and LS Columns (Miltenyi Biotec; Catalog number 130-042-401) was performed according to the manufacturer's instructions. Enriched CD4 SP thymocytes, mostly consisted of CD4^+^CD25^−^Foxp3^−^ cells, were stimulated in culture with anti-CD3, anti-CD28, interleukin-2 (IL-2) and transforming growth factor-beta (TGF-β), adapting a pre-established protocol for the differentiation of peripheral Treg cells (Fantini et al., 2007). Briefly, 96-well plates were coated with 5 μg/mL anti-CD3 antibody (Supplementary Table 1) for 90 min at 37 °C, the excess was removed, and the plate was left to dry. Thereafter, 2 × 10^5^ CD4^+^ T cells were added to complete RPMI 1640 (Gibco™) with 2 μg/mL anti-CD28, 1 μL/mL recombinant mouse IL-2 and 2 ng/mL recombinant mouse TGF-β. Approximately one million cells were separated for flow cytometry staining, which allowed checking the purity after the magnetic sorting. After 72 h incubation at 37 °C, the cells under differentiation were collected and stained for flow cytometry analysis, as described in the section above. Antibodies and cytokines used in these experiments are listed in Supplementary Table 1.

### *2.9* *In vitro* suppression assay

Thymic single-cell suspensions were prepared by mechanical disruption of the tissues through a 70-μm mesh in phosphate-buffered saline (PBS) (Gibco™). Cells were stained with viability dye for 20 min at room temperature, followed by surface antibody stains for 30 min at 4 °C, then washed with 2% FBS (Gibco™) in PBS. Cells were suspended in 2% FBS/PBS for cell sorting in a FACS Aria II device (BD Biosciences), in which CD4^+^ T cells were separated by their CD25 and CD45RB expression, yielding CD4^+^CD25^+^CD45RB^low^ regulatory T cells (Treg) and CD4^+^CD25^−^CD45RB^+/high^ conventional T cells (Tconv). Tconv cells were stained using the CFSE - Cell Labeling Kit (Abcam) for 5 min at room temperature. The cells were then seeded at different ratios (1:1, 1:2, 1:4, 1:8, and 1:16) and Tconv were stimulated with Dynabeads® Mouse T-Activator CD3/CD28 (Gibco™) on a 96-well plate. As a reference for autologous proliferation of CFSE stained cells, Tconv were seeded separately without coculture. Suppression capacity was analyzed 72 h later by investigating proliferating CD4^+^CD25^−^CD45RB^+/high^ cells via flow cytometry. Antibodies used in these experiments are listed in Supplementary Table 1.

### 2.10 TREC quantification

Thymic function was assessed by quantifying DβJβ T-cell receptor excision circles (TREC) and signal joint TREC (sjTREC) using real-time quantitative PCR (qPCR), following the methodology adapted from Dulude et al. (2008). The total sjTREC content was estimated by measuring δREC1 rearrangements with Jα61 and Jα58 segments, which together account for nearly 100% of sjTREC frequency in mice (Dulude et al., 2008; Verschuren et al., 1997). DβTREC were quantified by assessing Dβ1 rearrangements with Jβ1.1 to Jβ1.6 segments (Dulude et al., 2008). Genomic DNA was extracted from thymus, spleen, and inguinal lymph nodes using the DNeasy® Blood & Tissue Kit (Qiagen), according to the manufacturer's instructions (Document version April 2016). Extracted DNA served as template for an initial PCR preamplification using outer primers (Supplementary Table 2), where sj- and DβJβ-TREC were co-amplified alongside the CD4 gene, employed as an internal control. Preamplification was conducted on a T100™ Thermal Cycler (Bio-Rad) under the following conditions: initial denaturation at 95 °C for 10 min, followed by 22 cycles of 95 °C for 30 s, 60 °C for 30 s, and 72 °C for 2 min, concluding with a final elongation at 72 °C for 10 min and cooling to 20 °C. Subsequent quantification of TREC and CD4 was performed in duplicate via real-time qPCR using inner primers and SYBR® Green PCR Master Mix (Applied Biosystems) on a QuantStudio™ 3 Real-Time PCR System (Applied Biosystems). The qPCR cycling parameters included an initial denaturation at 95 °C for 5 min, followed by 40 amplification cycles of 95 °C for 10 s, 60 °C for 15 s, and 72 °C for 10 s. TREC copy numbers were normalized to the endogenous reference gene by calculating the ΔCt values: ΔCt = Ct (gene of interest; sjTREC or DβTREC) – Ct (housekeeping gene; CD4). The sj/βTREC absolute ratio was thus calculated as the sum of sj61 and sj58 TREC ΔCt, divided by the sum of DJβ1TREC ΔCt (Dion et al., 2007; Dulude et al., 2008). Primers used in these experiments are listed in Supplementary Table 2.

### 2.11 Linear Discriminant Analysis

To assess multivariate differences between WT and mSOD1, we applied Linear Discriminant Analysis (LDA) using the LDA function from the MASS package in R. LDA constructs a single discriminant score (LD1) as a linear combination of variables, chosen to maximize separation between predefined groups. Prior to analysis, all variables were standardized (z-scored), and the discriminant axis was oriented such that higher LD1 values correspond to WT and lower values to mSOD1. For reproducibility, the final LD1 equations, expressed in original measurement units (raw coefficients and intercept), are provided in supplementary Method 2.

### 2.12 Statistical analysis

Statistical comparisons between two groups were performed using unpaired Student's *t*-test in GraphPad Prism 10 software (Dotmatics). For experiments including multiple ages, comparisons were restricted to the same cellular population (or parameter) between age-matched WT and mSOD1 mice, with no statistical comparisons performed across different ages. The results were expressed as mean ± standard error (SE). Proportions were compared using a chi-square test for equality of proportions with continuity correction. For the spatial statistics, Ripley's L(r)−r function was computed for each sample, and the mean ± standard deviation (as envelopes of six replicates) was plotted for each genotype. To assess statistical differences between WT and SOD1 distributions, values of L(r)−r were compared at each distance r using Welch two-sample t-tests, with false discovery rate (FDR) correction for multiple testing. No significant differences were observed at any spatial scale, indicating that Treg clustering was similar in both genotypes. Sample sizes (n) for each experiment are reported in the respective figure legends. The differences between the groups were considered statistically significant when p < 0.05 (*), p < 0.01 (**), p < 0.001 (***) or p < 0.0001 (****).

## 3 Results

### 3.1 mSOD1 mice have decreased number of regulatory T cells in the thymus at the late stage of ALS

We first analyzed the thymus of mSOD1 (SOD1-G93A) mice at four different time points: 30 and 60 days-old, which correspond to a pre-symptomatic phase of ALS; 90 days-old, which, in this strain corresponds approximately to the onset of the disease with the beginning of the paralysis; and 120 days-old, the late-stage of ALS, in which most of the animals present advanced paralysis (Fig. 1A). We initially analyzed thymic alterations in both male and female mSOD1 mice throughout disease progression. Since the most prominent changes were observed in males, we focused the subsequent mechanistic analyses on male mice. The human mutated SOD1 (SOD1) protein was clearly expressed in the thymus of mSOD1, as observed by western blotting (Supplementary Fig. S1A), as well as the human SOD1 mRNA transcript, revealed through *in situ* hybridization (Supplementary Fig. S1B). We observed a significant reduction in the relative thymus weight of male mSOD1 at 120 days-old, corresponding to an approximately 24% decrease relative to WT controls (p = 0.0003; Fig. 1B). Interestingly, mSOD1 mice have higher relative thymus weight in the pre-symptomatic phase of ALS than age-matched WT controls (30 days-old), whereas no differences were observed at 60 or 90 days-old (Fig. S1C). The relative thymus weight becomes significantly lower after the onset of the disease, at the late-stage (120 days-old), as previously shown in Fig. 1B. In addition to the decrease in the thymus weight, male mSOD1 mice exhibited a significant reduction in total thymocyte numbers, corresponding to an approximately 40% decrease compared with WT controls, at 120 days-old (p = 0.003; Fig. 1C).

**Fig. 1:**
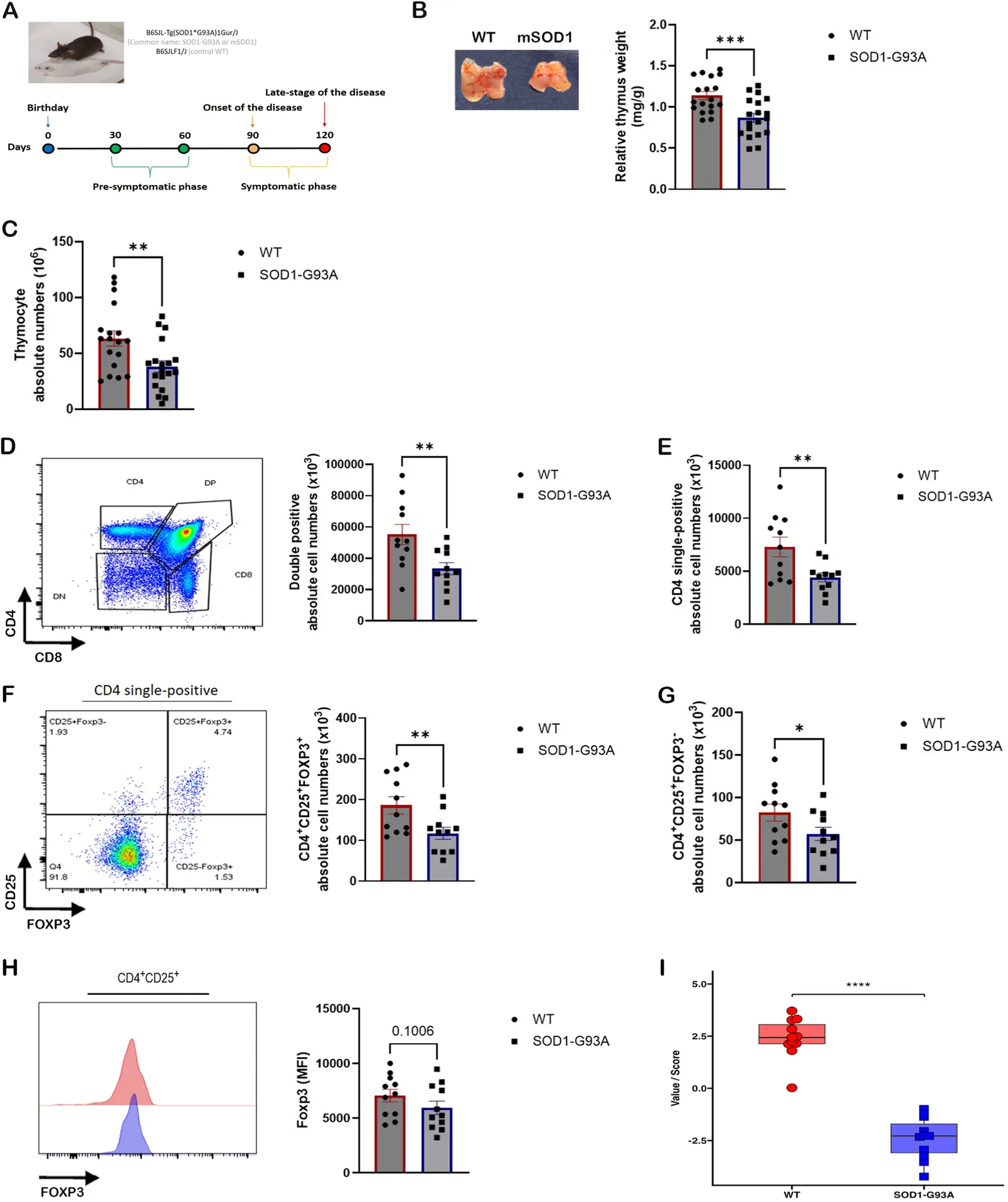
**mSOD1 mice have decreased number of regulatory T cells in the thymus at late stage of ALS**. The scheme in **(A)** shows the different mouse strains and time points analyzed in the present study. **(B)** Representative picture and macroscopy and relative thymus weight of B6SJLF1/J control (WT; red/gray bar) and mSOD1 (SOD1-G93A; blue/gray bar) male mice at 120 days-old. **(C)** The graph shows the thymocyte absolute number of WT (red/gray bar) and SOD1-G93A (blue/gray bar) mice. **(D)** Panel on the left shows a representative dotplot and the gate strategy used to define the different thymocyte subpopulations based on the CD4 and CD8 co-receptor expression, analyzed by flow cytometry. DN = CD4^−^CD8^−^ double-negative; DP = CD4^+^CD8^+^ double-positive; CD4^+^ = CD4^+^CD8^−^ SP; CD8^+^ = CD4^−^CD8^+^ SP. The graph at right shows the DP absolute numbers in the WT (red/gray bar) and SOD1-G93A (blue/gray bar) mouse thymi. **(E)** The graph shows the CD4 SP absolute numbers in WT (red/gray bar) and SOD1-G93A (blue/gray bar) mouse thymi. **(F)** The panel at left shows a representative dotplot and the gate strategy used to identify the regulatory T cells (CD4^+^CD25^+^Foxp3^+^) and regulatory T cell progenitors (CD4^+^CD25^+^Foxp3^-^) based in the CD25 and Foxp3 expression in the cells expressing CD4. The graph on the right shows the regulatory T cell (CD4^+^CD25^+^Foxp3^+^) absolute numbers in the WT (red/gray bar) and SOD1-G93A (blue/gray bar) mouse thymi. **(G)** Absolute numbers of regulatory T cell progenitors (CD4^+^CD25^+^Foxp3^-^) numbers in the WT (red/gray bar) and SOD1-G93A (blue/gray bar) mouse thymi. **(H)** The panel shows a representative histogram of the Foxp3 median fluorescence intensity on CD4^+^CD25^+^ WT (red/gray bar) and SOD1-G93A (blue/gray bar) thymocytes and the graph shows its quantification. MFI = median fluorescence intensity. **(I)** Linear discriminant analysis (LDA) of thymocyte populations distinguishes WT (red) and mSOD1 mice (blue). Sample sizes were: (B–C) n = 18 WT and n = 19 mSOD1; (D–I) n = 11 WT and n = 11 mSOD1. Results are expressed as mean ± SEM and were analyzed by unpaired Student's *t*-test. Differences were considered statistically significant when * p < 0.05, **p < 0.01, ***p < 0.001 and ****p < 0.0001. (For interpretation of the references to color in this figure legend, the reader is referred to the Web version of this article.)

We next assessed the different thymocyte subpopulations found during T cell maturation and differentiation. According to the thymic atrophy observed in 120 days-old, we found a significant decrease in the DP thymocytes absolute number in male mSOD1 representing an approximately 40% reduction compared with WT mice (p = 0.0044; Fig. 1D), while no significant difference was observed in the DP relative numbers at the same conditions (Fig. S1D).

Regarding mature conventional and regulatory T (Treg) cells, we observed reductions in the absolute numbers of CD4 SP cells (approximately 40% decrease; p = 0.0049), CD4^+^CD25^+^Foxp3^+^ Treg cells (approximately 37% decrease; p = 0.0075), and CD4^+^CD25^+^Foxp3^-^ Treg progenitors (approximately 30% decrease; p = 0.0315) in male mSOD1 mice compared with WT controls (Fig. 1E–G; Supplementary Fig. S1E–G). No difference was observed in the density of Foxp3 expression in the mSOD1 CD4^+^CD25^+^ cells, when compared to the corresponding WT controls (Fig. 1H). Of note, the mSOD1 mice at 60 days-old have an inversed profile, with approximately 72% higher thymic Treg numbers than WT littermate controls (p = 0.003), whereas there is no difference at 30 or 90 days-old (Supplementary Fig. S1H). Interestingly, as seen in Supplementary Fig. S1I, the thymic atrophy seen in more advanced stages of the disease, occurred in the absence of a significant rise in circulating corticosterone, widely known to induce thymic involution (Savino et al., 2015).

Among other thymocyte subpopulations, male mSOD1 mice presented an increase in total DN relative numbers compared with WT controls (5.3% vs 4.3%, respectively; p = 0.0345) (Fig. S2A), while absolute numbers of the most immature DN1 cells were decreased by approximately 37% (p = 0.0348; Fig. S2B).

No differences were observed in the number of CD8 single positive or CD8^+^CD28^low^ cells (Supplementary Fig. S2C–S2D), which were previously identified to have suppressive capacity in the thymus (Vuddamalay et al., 2016).

We further subdivided the CD4 and CD8 SP cells, as a function of CD69 and CD62L expression. CD69 is transiently expressed on activated mature T cells and on thymocytes undergoing positive or negative selection in the thymus. Its expression prevents migration of less mature CD69^+^CD62L^−^ SP cells out of the thymus, being its negative regulation necessary for egress (Feng et al., 2002; Nakayama et al., 2002; Yamashita et al., 1993). SP thymocytes at the final stage of maturation loose CD69 and upregulate CD62L, becoming CD69^−^CD62L^+^ cells that are therefore ready to leave the thymus towards the periphery (Xu and Ge, 2014). We observed a reduction in the CD4^+^CD69^+^CD62L^−^ SP cell numbers in mSOD1 mouse thymus, while the other CD4 and CD8 subpopulations presented no alterations, compared with the counterpart subpopulations seen in WT mice (Supplementary Fig. S2E–S2H).

Linear discriminant analysis (LDA) was performed using multiple parameters, including ROW, total cell count, DN (DN1-DN4), DP, CD4 SP, CD8 SP, Treg, and CD4^+^CD25^+^Foxp3^-^ Treg progenitor absolute cell numbers, besides CD69 *versus* CD62L-defined subsets and Foxp3 MFI in CD4^+^CD25^+^ cells. The analysis revealed a significant separation between WT and mSOD1 mice at 120 days-old (Fig. 1I).

Female mSOD1 presented different kinetics, with relative thymus weight significantly lower than the WT controls at the onset of ALS (90 days-old) and slightly higher at the late stage (120 days-old; Supplementary Fig. S3A). Moreover, no significant reduction was observed in the total thymocyte cell count (Supplementary Fig. S3B), in the relative and absolute numbers of DP (Supplementary Fig. S3C), or in relative and absolute numbers of SP CD4 thymocytes (Supplementary Fig. S3D) at 120 days old. However, relative and absolute numbers of Treg were considerably decreased in late stage mSOD1 mice, when compared with littermate controls (Supplementary Fig. S3E). No significant alteration in circulating corticosterone was observed in female mSOD1 mice (Supplementary Fig. S3F). Furthermore, male mSOD1 mice expressed higher levels of mutant human SOD1 in the thymus, as quantified by western blotting (Supplementary Fig. S3G). Therefore, subsequent analyses focused on 120-day-old male mice, in which thymic alterations were more robust and reproducible.

We investigated the expression of the integrins CD49d and CD49e (the α4 and α5 integrin chains of the fibronectin receptors VLA (very late antigen)-4 and VLA-5, respectively), and CD49f (the α6 integrin chain of the laminin receptor VLA-6), in addition to the CXCL12 receptor, CXCR4, and the sphingosine-1-phosphate (S1P) receptor 1 (S1P1) in the Treg cells of mSOD1 mice. The S1P1 is the major receptor involved in the egress of T cells from the thymus to peripheral lymphoid organs (Allende et al., 2004; Matloubian et al., 2004). As shown in the Supplementary Fig. S4A, there is a decrease in the Treg cells expressing those receptors that is in accordance with the decrease in the Treg cell number in mSOD1 mice compared to the WT, although no difference in the density of receptor expression was observed, as ascertained by the median fluorescence intensity (Supplementary Fig. S4A).

### 3.2 mSOD1 mice have decreased numbers of mature medullary TEC at the late stage of ALS

As mentioned before, thymocyte differentiation occurs as cells migrate within the thymic lobules. Developing thymocytes encounter cortical and medullary microenvironments, through distinct cell–cell and cell–matrix interactions. These interactions allow thymocytes to migrate into the thymic parenchyma and interact with the appropriate microenvironment at each stage of their development (Savino et al., 2002, 2004). We thus investigated the TEC network in mSOD1 mice, aiming to understand whether the cortico-medullary compartmentalization is preserved. As seen in Fig. 2A–B, by immunofluorescence staining of thymic medullary (CK5) and cortical (CK8) cytokeratins, there is a clear definition of cortex and medulla in WT mice, while in mSOD1 although overall preserved, some medullary areas branched to the cortex. The ratio between the total epithelium-free, the cortical and the medullary areas and the total thymus area remained similar between the two groups (Fig. 2A–B and Fig. 1B). We observed by flow cytometry a decrease in the cortical CD45^−^EpCam^+^Ly51^+^UEA1^−^ TEC (cTEC; approximately 34% decrease) and in the medullary CD45^−^EpCam^+^Ly51^−^UEA1^+^ TEC (mTEC; approximately 27% decrease), as well as a significant reduction in the total number of EpCAM^+^ TEC (approximately 31% decrease; p = 0.0478) and in mature mTEC, expressing high amounts of CD80 and class II major histocompatibility complex (MHC) molecule (CD45^−^EpCam^+^Ly51^−^UEA1^+^CD80^high^MHCII^high^) (approximately 32% decrease; p = 0.0322) in mSOD1 mouse thymus when compared with the corresponding populations in WT mice (Fig. 2C–G).

**Fig. 2:**
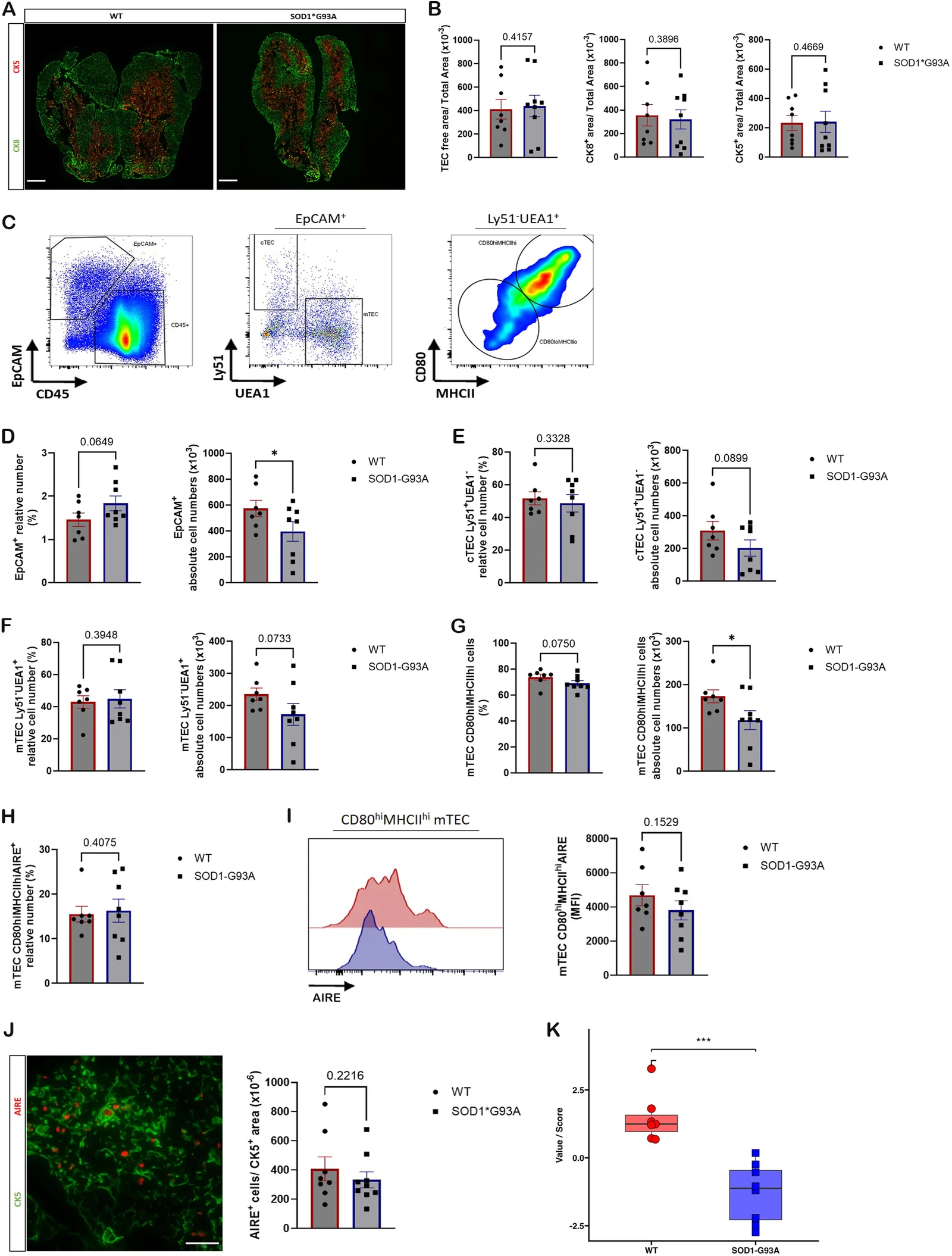
mSOD1 mice have decreased number of mature medullary thymic epithelial cells at late stage of ALS. **(A)** Representative immunohistochemistry of cytokeratin 8 (CK8) and cytokeratin 5 (CK5) expression profile in entire slices of thymi from WT or SOD1-G93A mice, showing cortical (CK8^+^) and medullary (CK5^+^) regions; scale = 1 mm. (**B**) Quantification of the TEC-free area (left), cortical area (middle, CK8^+^), and medullary area (right, CK5^+^) relative to total thymic area in WT (red/dark gray bars) and SOD1-G93A (blue/light gray bars) thymus sections. **(C)** Representative panels showing the gate strategy to define the total EpCAM^+^ TEC (left), EpCAM^+^Ly51^+^UEA1^-^ cortical *versus* EpCAM ^+^ Ly51^−^UEA1^+^ medullary TEC (middle) and EpCAM^+^Ly51^−^UEA1^+^CD80^high^MHCII^high^ mature mTEC (right). **(D-G)** Graphs show the relative and absolute numbers of total EpCAM^+^ epithelial cells **(D)**, cortical EpCAM^+^Ly51^+^UEA1^-^ cells **(E)**, medullary EpCAM^+^Ly51^−^UEA1^+^ cells **(F)** and mature medullary EpCAM^+^Ly51^−^UEA1^+^CD80^high^MHC-II^high^ cells **(G)** in the WT (red/gray bar) and SOD1-G93A (blue/gray bar) mouse thymi. **(H)** Relative number of Aire^+^ mature medullary EpCAM^+^Ly51^−^UEA1^+^CD80^high^MHCII^high^ cells in the WT (red/gray bar) and SOD1-G93A (blue/gray bar) mouse thymi. **(I)** The panel shows a representative histogram of the Aire median fluorescence intensity on mature medullary EpCAM^+^Ly51^−^UEA1^+^CD80^high^MHCII^high^ cells in the WT (red/gray bar) and SOD1-G93A (blue/gray bar) thymus and the graph at right shows its quantification. **(J)** Immunofluorescence showing Aire (red) and CK5 (green) expression in thymus sections; scale bar = 100 μm. The graph shows the number of Aire^+^ cells per medullary (CK5^+^) area in WT (red/gray bars) and SOD1-G93A (blue/gray bars) thymus sections. **(K)** Linear discriminant analysis (LDA) of thymocyte populations distinguishes WT (red) and mSOD1 mice (blue). For histological analyses (A, B and J), n = 8 WT and n = 9 mSOD1 mice were analyzed, with four thymic sections per mouse collected at fixed 200-μm intervals. For flow cytometry and LDA analyses (C–I and K), n = 7 WT and n = 8 mSOD1 mice. Results are expressed as mean ± SEM and were analyzed by unpaired Student's *t*-test. Differences were considered statistically significant when * p < 0.05 and ***p < 0.001. (For interpretation of the references to color in this figure legend, the reader is referred to the Web version of this article.)

Given the reduction in mature mTEC numbers, we next assessed the autoimmune regulator (Aire) expression. No differences were observed in the relative numbers and in the density of Aire expression by the mature mTEC or in the number of Aire^+^ cells detected per total thymus area in mSOD1 mice (Fig. 2H–J). LDA performed on TEC parameters, including ROW, total cell count, EpCAM^+^, cTEC, mTEC, and mature mTEC absolute cell numbers (besides Aire MFI in mature mTEC), revealed a significant separation between WT and mSOD1 mice, indicating marked alterations in the thymic epithelial compartment (Fig. 2K).

### 3.3 Thymi of mSOD1 mice present abnormally higher numbers of regulatory T cells at large distances from the medullary TEC at late stage of ALS

We analyzed the distribution of Foxp3^+^ Treg cells in relation to CK5^+^ mTEC in thymic sections (Fig. 3A). Most Treg were in intimate contact with mTEC, in a distance lower than 10 μm from each other, in both control and mSOD1 mice (Fig. 3B–C), as expected. Although representing only a minor fraction of the total Treg population, the proportion of Treg cells located at large distances from mTEC was higher in mSOD1 mice than in WT controls (Fig. 3B–C). As seen in Fig. 3C–D, although mSOD1 Foxp3 positive immunostaining was concentrated mainly in the medulla, Foxp3^+^ cells were sparse and separated from CK5^+^ mTEC and abnormally present in the thymic CK5^-^ cortical region within the thymus (Fig. 3C–D). In parallel, thymi of mSOD1 mice present an increased area of FN in both cortical (approximately 41% increase; p = 0.0128) and medullary (approximately 58% increase; p = 0.0279) regions, when compared to littermate controls (Supplementary Fig. S4B–D).

**Fig. 3:**
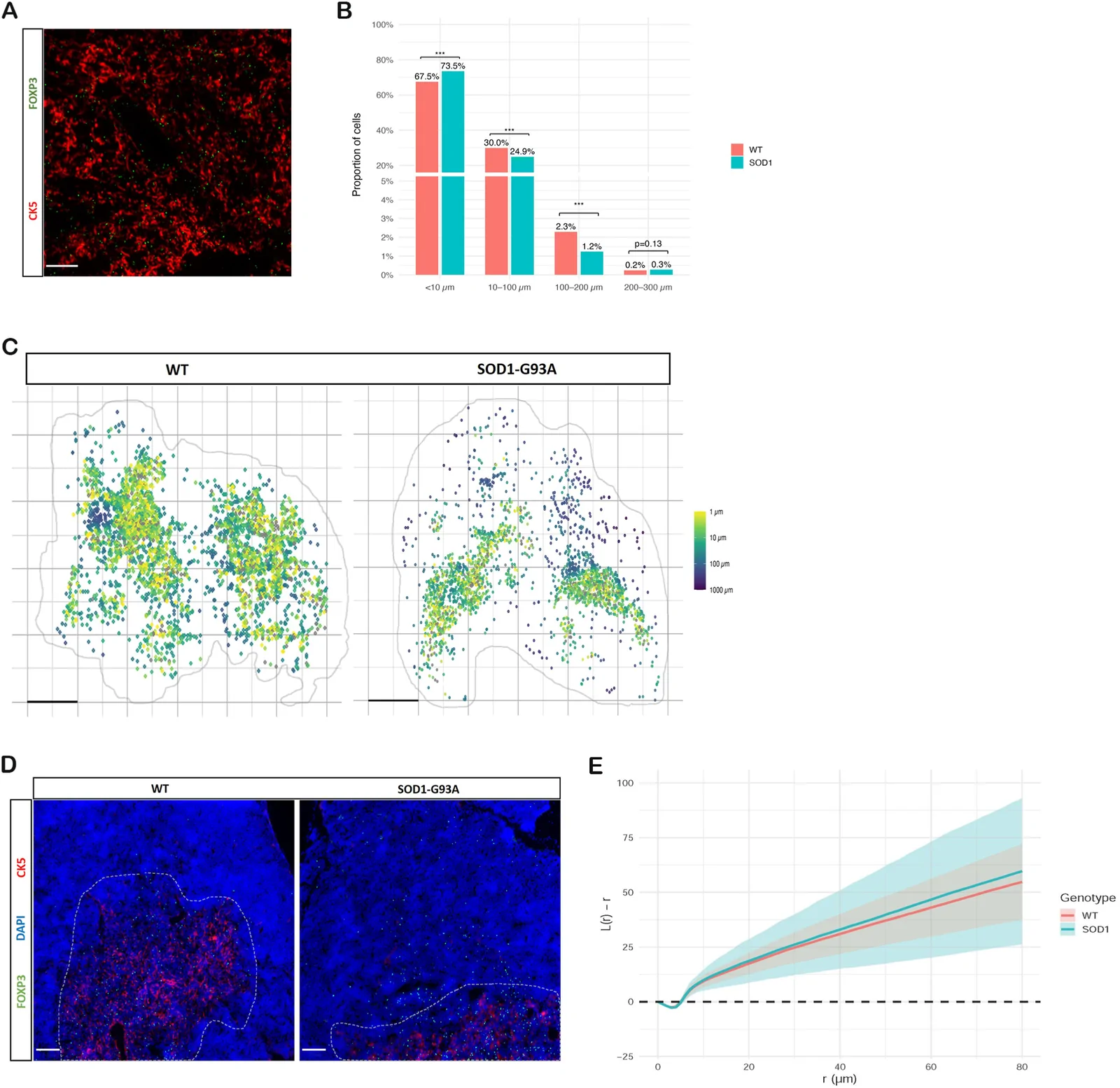
**mSOD1 present higher numbers of regulatory T cells at large distances from the medullary epithelial cells in the thymus at late stage of ALS**. **(A)** Immunohistochemistry showing the Foxp3 and cytokeratin 5 (CK5) expression profile in the thymus; scale = 100 μm. **(B)** Spatial distribution of cells relative to CK5^+^ cells in WT and SOD1-G93A samples. Bar plots represent the proportion of cells located at increasing distances from CK5^+^ cells in WT (red) and mSOD1 (blue) tissues. Bars indicate mean percentages; numbers on top of each bar represent the proportion of cells. **(C)** Spatial localization of Foxp3^+^ cells relative to CK5^+^ cells in WT (left) and SOD1-G93A (right) thymus sections. Each dot represents one Foxp3^+^ cell, stained according to –log_10_ (distance to the nearest CK5^+^ cell), with gray dots indicating direct contact (distance = 0). Cooler colors indicate closer proximity (1–10 μm), whereas warmer colors represent increasing distances (up to 1000 μm). Scale bar = 1 mm. **(D)** Representative immunofluorescence of the DAPI, Foxp3 and cytokeratin 5 (CK5) expression profile in the thymus of WT and SOD1-G93A mice showing cortical (CK5^-^) and medullary (CK5^+^; gray dashed lines) regions; scale = 100 μm. **(E)** Curves represent the mean Ripley's L(r)–r per genotype (WT in blue, SOD1 in red). The colored shading around each curve indicates the standard deviation (SD) of 6 individual mouse curves. At each radius r (0–80 μm), a Welch two-sample *t*-test was performed between genotypes, and *p*-values were adjusted for multiple testing using the Benjamini–Hochberg false discovery rate (FDR) procedure. Shaded vertical bands indicate radii with FDR-adjusted p < 0.05; in this dataset, no radius reached significance after correction. All histological and spatial analyses (A–E) were performed using thymic sections from n = 6 WT and n = 6 mSOD1 mice, with four sections per mouse collected at fixed 200-μm intervals. (For interpretation of the references to color in this figure legend, the reader is referred to the Web version of this article.)

Ripley's L function analysis revealed that both WT control and mSOD1 Foxp3^+^ cells were clusterly distributed in the thymus lobules, which is consistent with the fact that the majority of the Treg cells are in close contact with mTEC and are therefore found in the thymic medullary region (Fig. 3E).

### 3.4 mSOD1 mouse Treg cells express higher expression of apoptosis marker in the thymus and are dysfunctional

We next investigated whether the abnormal localization of mSOD1 Foxp3^+^ Treg cells might be associated with alterations in their maturation process. Using annexin V staining by flow cytometry, we observed an increase in the apoptosis rate in the mSOD1 thymocytes (1.28-fold increase; p = 0.0038), as well as in the CD4 SP cells (1.30-fold increase; p = 0.0015) and in the CD4^+^CD25^+^ Treg precursor compartment (1.84-fold increase; p = 0.0017), when compared to the corresponding WT control populations (Fig. 4A–C).

**Fig. 4:**
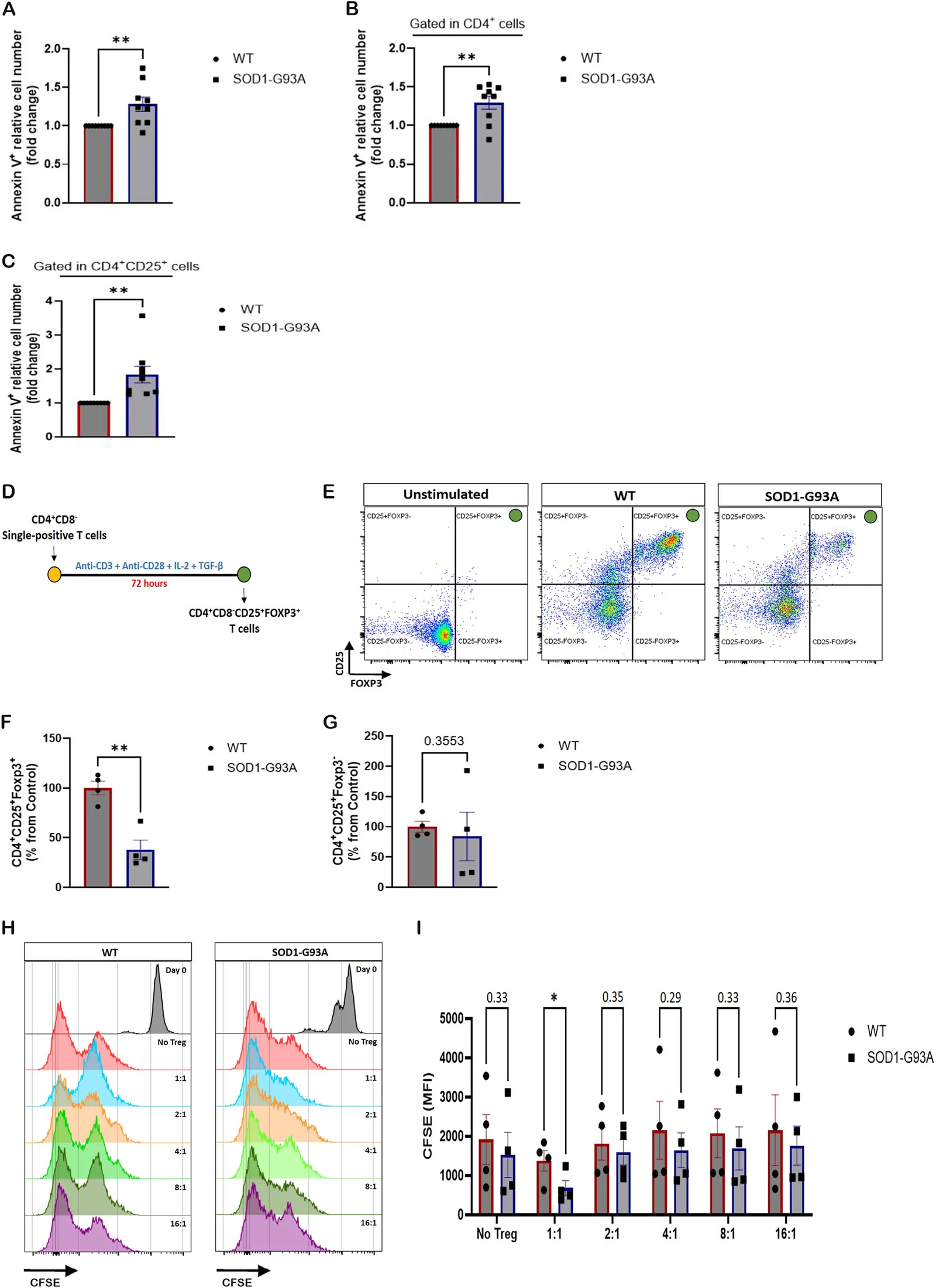
**mSOD1 mice show increased apoptosis in the thymus and impaired *in vitro* differentiation and function of regulatory T cells at late stage of ALS**. **(A)** The graph shows annexin V^+^ cells in the total SOD1-G93A (blue/gray bar) mouse thymi compared to the WT control group (red/gray bar). Data are shown as fold change related to the control WT animals. **(B)** The graph shows annexin V^+^ cells in the SOD1-G93A (blue/gray bar) CD4 single positive population compared to the WT control group (red/gray bar) as a fold change. **(C)** The graph shows annexin V^+^ cells in the SOD1-G93A (blue/gray bar) CD4^+^CD25^+^ compartment compared to the WT control group (red/gray bar) as a fold change. **(D)** The scheme represents the strategy used to induce isolated CD4^+^CD8^−^ SP thymocytes to differentiate into CD4^+^CD8^−^CD25^+^Foxp3^+^*in vitro*. **(E)** Representative dotplots showing the CD25 *versus* Foxp3 populations obtained after 72 h *in vitro* with or without stimulation. **(F-G)** Graphs showing the **(F)** CD4^+^CD25^+^Foxp3^+^, and the **(G)** CD4^+^CD25^+^Foxp3^-^ cells, after 72 h stimulation with anti-CD3/CD28 + IL-2 + TGF-β. SOD1-G93A cell differentiation (blue/gray bar) is expressed as percentage of the WT control (red/gray bar). **(H)** Representative histograms of CFSE fluorescence on CD4^+^CD25^−^CD45RB^+/high^ effector T cells from WT and SOD1-G93A mice at day 0 and after 72 h under anti-CD3/CD28 stimulation. **(I)** The graph shows the CFSE median fluorescence intensity on CD4^+^CD25^−^CD45RB^+/high^ WT (red/gray bar) and SOD1-G93A (blue/gray bar) T cells after 72 h under anti-CD3/CD28 stimulation, with no CD4^+^CD25^+^CD45RB^low^ regulatory T cells (Treg) or with the presence of Treg at different ratios. Treg = CD4^+^CD25^+^CD45RB^low^. MFI = median fluorescence intensity. For apoptosis analyses (A–C), n = 9 WT and n = 9 mSOD1 mice. For *in vitro* Treg differentiation and suppression assays (D–I), n = 4 WT and n = 4 mSOD1 mice. Results are expressed as mean ± SEM and were analyzed by unpaired Student's *t*-test. Differences were considered statistically significant when * p < 0.05 and **p < 0.01. (For interpretation of the references to color in this figure legend, the reader is referred to the Web version of this article.)

To understand the Treg cell-intrinsic effect of SOD1 mutation, we performed an *in vitro* differentiation assay where enriched CD4 SP thymocytes, were stimulated in culture to differentiate into Treg cells (Fig. 4D). After 72 h, WT CD4 SP thymocytes induced close to 100% of CD4^+^CD25^+^Foxp3^+^ Treg differentiation. By contrast, mSOD1 CD4 SP thymocytes showed reduced Treg differentiation capacity under the same conditions, generating approximately 62% fewer Treg cells than WT controls (p = 0.001), which was not related to a decreased ability to reach the Treg precursor stage (Fig. 4E–G). These results show that mSOD1 thymocytes have an intrinsically poor capacity to differentiate into Treg cells.

We also observed the Treg suppressive capacity by measuring the ability of Treg to suppress conventional T cell (Tconv) proliferation *in vitro*. Briefly, to induce suppression, CD4^+^CD25^+^CD45RB^low^ Treg cells and CFSE-stained CD4^+^CD25^−^CD45RB^+/high^ Tconv cells were co-cultured in different ratios with addition of anti-CD3 and anti-CD28 activation beads. After 72 h, the proliferation of the Tconv population was analyzed by CFSE dilution as a read-out to assess the extent to which the Treg cells can suppress Tconv cell activation (Collison and Vignali, 2011). A representative experiment is shown in Fig. 4H, in which mSOD1 Tconv cells presented lower CFSE MFI when compared to the WT cells, indicating higher proliferation in all the conditions tested, being significantly different at the 1:1 ratio, where cultures containing mSOD1-derived Treg displayed approximately 50% lower CFSE MFI than cultures containing WT-derived Treg (p = 0.038; Fig. 4I). These data indicate that, despite the Foxp3 expression, mSOD1-isolated Treg cells have lower ability to exert suppressive function in the tested conditions.

### 3.5 mSOD1 mice have alteration in the final stage of thymocyte maturation and reduced Treg numbers in draining lymph nodes

Aiming to understand whether the observed thymic environment alterations and the abnormal thymocyte intrathymic spatial localization could affect the T cell maturation and export, we next examined the T-cell receptor (TCR) excision circles (TREC) within the thymus and in the periphery. Specifically, βTREC are generated during TCRβ chain recombination at the CD4^−^CD8^−^ double-negative 3 (DN3) stage, while signal joint TREC (sjTREC) originate from the recombination process of TCRα at the double-positive (DP) stage, due to the genomic organization of the TCR loci, where the TCRδ locus is embedded within the TCRα locus (de Villartay et al., 1988), as seen in Fig. 5A. We observed that βTREC levels did not differ between mSOD1 and WT thymi. However, mSOD1 mice did exhibit an approximately 10% reduction in intrathymic sjTREC levels compared to the WT controls (p = 0.0381; Fig. 5B). Yet, the analysis in the periphery revealed that mSOD mice have similar sjTREC/βTREC absolute ratio in the spleen and inguinal lymph nodes (iLN) when compared to the controls (Supplementary Fig. S5A).

**Fig. 5:**
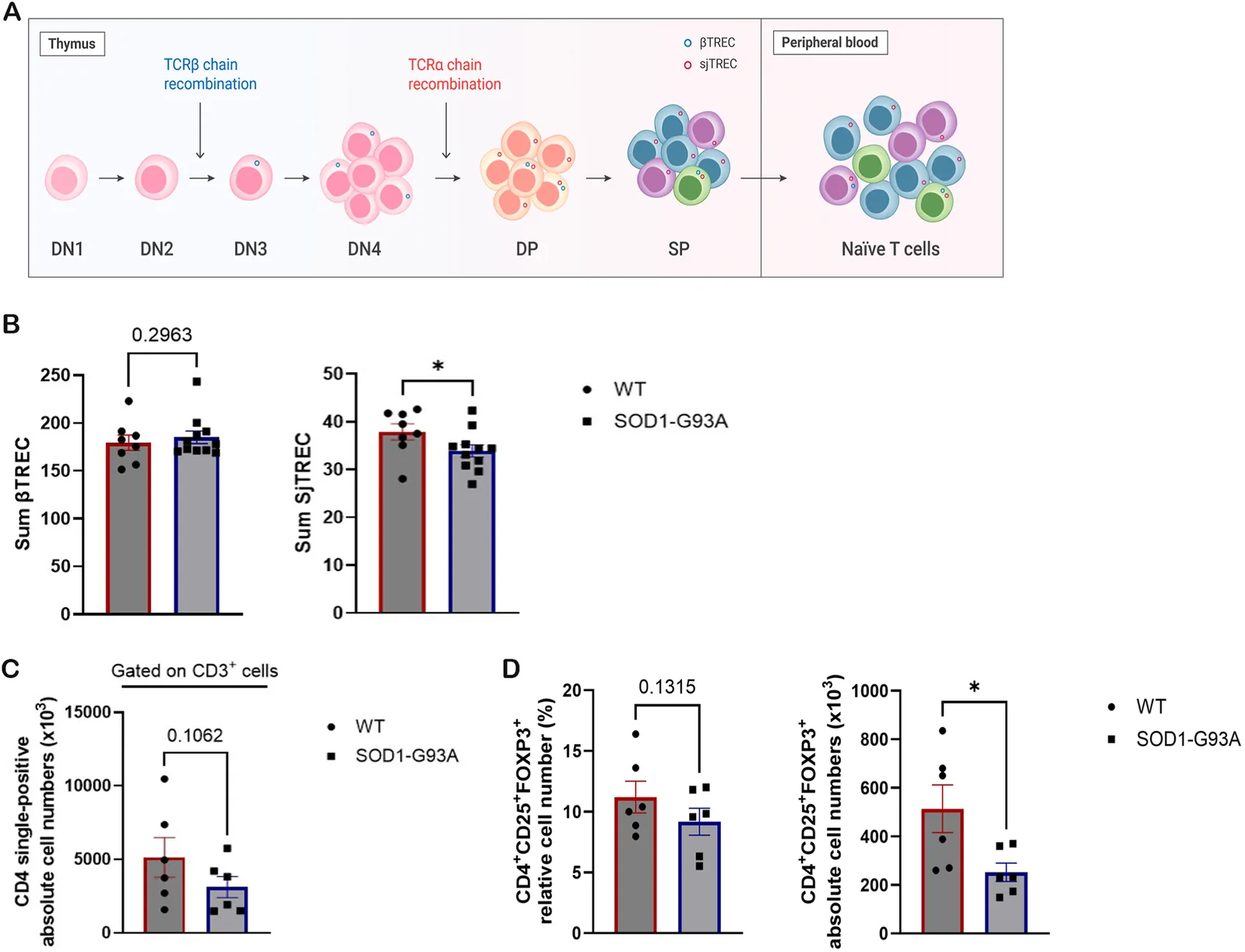
**mSOD1 mice have impaired thymocyte differentiation and decreased levels of Treg in the inguinal lymph nodes at late stage of ALS**. **(A)** Scheme representing generation of βTREC and sjTREC, during β and α chain TCR recombination, respectively, throughout thymocyte differentiation. DN = double-negative; DP = double-positive; SP = single-positive. **(B)** The graphs show the sum of DJβ1TREC (βTREC; left) and sj61 and sj58 (sjTREC; right) of WT (red/gray bar) and SOD1-G93A (blue/gray bar) mouse thymi. **(C)** The graph shows the CD4 SP absolute numbers in WT (red/gray bar) and SOD1-G93A (blue/gray bar) mouse iLN. **(D)** The graphs show the regulatory T cell (CD4^+^CD25^+^Foxp3^+^) relative (left) and absolute (right) numbers in the WT (red/gray bar) and SOD1-G93A (blue/gray bar) mouse iLN. TREC quantification (B) was performed in n = 8 WT and n = 11 mSOD1 mice. Inguinal lymph node (iLN) analyses (C–D) were performed in n = 6 WT and n = 6 mSOD1 mice. Results are expressed as mean ± SEM and were analyzed by unpaired Student's *t*-test. Differences were considered statistically significant when * p < 0.05. (For interpretation of the references to color in this figure legend, the reader is referred to the Web version of this article.)

Finally, we found that the number of Treg cells is reduced in the periphery of mSOD1 mice compared to the WT counterparts, which is particularly prominent in the inguinal lymph nodes involved in the lymphatic drainage of the hindlimbs (approximately 51% fewer Treg cells than WT controls; p = 0.0162), although the ratio between Treg and effector T cells (Treg/Teff) and the level of Foxp3 expression in the Treg were preserved (Fig. 5C–D and Supplementary Fig. S5B–F). This reduction in the Treg cell contents is in line with what we observed in the thymus of mSOD1 mice and to what has been described in the peripheral blood of patients during the late stage of ALS (Beers et al., 2017).

## 4 Discussion

Our results support and expand the concept that ALS is a “non-cell autonomous” disease, in which neuronal degeneration occurs in the context of complex interactions between neural, glial and immune cells (Boillée et al., 2006; Di Giorgio et al., 2007; Philips and Rothstein, 2014; Van Harten et al., 2021). Using the SOD1-G93A mouse model (mSOD1), we demonstrate that the thymus is affected during disease progression, suggesting that alterations in central T-cell development may contribute to the immune dysfunction associated with ALS. We showed that mSOD1 mice at 120 days exhibit decreased thymus weight and total thymocyte numbers, particularly affecting double-positive (DP) thymocytes, CD4 single-positive (SP) and Treg cells. Decreased Treg and Treg progenitors, combined with unchanged Foxp3 density, indicates that mutant SOD1 affects cell number rather than Foxp3 expression, which aligns with reports that Treg functionality can be impaired without loss of Foxp3, such as in inflammatory environments (Josefowicz et al., 2012). These changes suggest that ALS may undermine the body's ability to generate and maintain protective immune cells, fueling further imbalance. Moreover, we observed a strikingly similar trajectory within the thymus compared to what is observed in ALS patients' peripheral blood (Beers et al., 2017): thymic Treg numbers were elevated during early disease stages (60 days-old) but diminished at late stages (120 days-old) in mSOD1 mice. It is important to consider that the earliest time points analyzed in this study (30 and 60 days of age) correspond to young mice prior to the onset of physiological age-related thymic involution, a period during which thymic cellularity remains high and the organ has not yet undergone the progressive decline observed later in life (Gui et al., 2012; Palmer, 2013; Shanley et al., 2009). Therefore, observations made at these pre-symptomatic stages should be considered in light of the distinct biological characteristics of the thymus at these ages. Nevertheless, WT and mSOD1 mice were compared exclusively at the same ages, allowing genotype-associated alterations to be distinguished from age-dependent changes in thymic homeostasis.

Interestingly, thymic alterations were more pronounced in males, whereas female mSOD1 mice displayed a milder phenotype. This observation is consistent with previous reports of sex-dependent differences in ALS susceptibility and progression, with female mice frequently exhibiting delayed disease onset and slower disease progression compared with males (Choi et al., 2008; Heiman-Patterson et al., 2005). Such differences have been attributed, at least in part, to the neuroprotective and immunomodulatory effects of estrogens, as ovariectomy accelerates disease progression and shortens survival in female mSOD1 mice, whereas estradiol supplementation restores this protective phenotype (Choi et al., 2008). In addition, estradiol treatment has been shown to improve motoneuron survival and reduce neuroinflammatory signaling in male mSOD1 mice (Heitzer et al., 2017). Thymic atrophy is a well-documented consequence of chronic stress, aging, and neuroinflammatory conditions (Savino et al., 2007). The higher expression of mutant human SOD1 observed in the thymus of male mice further suggests that local cell-intrinsic effects of the transgene may contribute to the greater thymic impairment observed in males.

Furthermore, we observed a thymic microenvironment impairment in the ALS mouse model, with a decrease in total TEC numbers and in the CD80^high^MHC-II^high^ mature medullary TEC (mTEC), whereas Aire expression remained preserved. mTEC are essential for negative selection and Treg differentiation (Perniola, 2018). Reduction in mTEC likely impairs Treg selection and contributes to abnormal Treg numbers. Although preserved Aire expression suggests that central tolerance is not entirely lost, fewer mTEC may reduce antigen presentation diversity, leading to autoimmune risk or dysregulated Treg generation. Additionally, Foxp3^+^ Treg are found farther from mTEC, sometimes in cortical regions, in mSOD1 mice. Proper thymocyte-mTEC interaction is critical for Treg selection (Ohkura and Sakaguchi, 2010), since mislocated Treg may receive suboptimal TCR and cytokine signals, contributing to their intrinsic dysfunction and increased apoptosis. This may reflect migratory defects due to altered ECM deposition (increased fibronectin contents) or integrin signaling deficiencies, since integrin function can be impaired despite normal expression levels (Andrew et al., 1998; Hidalgo and Frenette, 2009; McDowall et al., 2003; Pouwels et al., 2012).

Also, ECM alterations in neurodegenerative models have been implicated in aberrant immune cell trafficking (Savino et al., 2002, 2004), suggesting a broader effect of ALS pathology on thymic architecture. Moreover, we observed increased apoptosis marker in the mSOD1 thymocytes, and *in vitro* differentiation assays showed that mSOD1 thymocytes have reduced Treg differentiation and suppressive function despite normal Foxp3 induction, suggesting the existence of cell-intrinsic defects in Treg, independent of the thymic microenvironment. The differentiation of Treg relies not only on T cell receptor (TCR) engagement but also critically depends on CD28-mediated costimulation. This signaling axis facilitates the upregulation of essential Treg markers such as FOXP3, GITR, and CTLA-4, while also enhancing IL-2 production, a cytokine vital for Treg cell viability and function (Burchill et al., 2008). Additionally, TGF-β plays a pivotal role in supporting Treg development and persistence by limiting apoptotic cell death (Burchill et al., 2008; Jurberg et al., 2015; Liu et al., 2008). Reduced differentiation capacity can be due to defective IL-2/TGF-β signaling or CD28 co-stimulation, essential for the maintenance of Treg cell numbers and function (Burchill et al., 2008). Yet, further analyses are necessary to test this hypothesis.

Increased levels of glucocorticoids lead to acute thymic atrophy and immature DP thymocytes are particularly sensitive to glucocorticoids-induced apoptosis (Gomez-Sanchez, 2009). Herein, we did not observe significant alteration in the circulating corticosterone levels in the mSOD1 mice compared to the WT controls, suggesting that the observed thymic involution and elevated thymocyte apoptosis occurred via corticosterone-independent mechanism(s) in the mSOD1 mice. One possible explanation involves alterations in the neuroimmune axis connecting the central nervous system and the thymus. Sympathetic innervation plays a key role in maintaining thymic architecture and thymocyte development, and disruption of this circuitry has been associated with thymic dysfunction in other neurological disorders. In experimental autoimmune encephalomyelitis, neurological inflammation was shown to alter thymic innervation, microenvironmental organization, and T-cell development, indicating impairment of the bidirectional thymus–CNS communication axis (Francelin et al., 2023). Likewise, in a mouse model of Krabbe disease, degeneration of autonomic fibers was proposed as a mechanism contributing to progressive thymic atrophy, lymphopenia, and loss of immune competence (Galbiati et al., 2007).

Altogether, our findings suggest that the thymus itself may be also a target organ in ALS, with its output of Treg both reflecting and potentially reinforcing the peripheral immune phenotype. Accordingly, thymic Treg dysfunction may impair peripheral immune regulation, contributing to the neuroinflammation observed in ALS (Beers et al., 2011; Thonhoff et al., 2018).

Our data also indicates that mSOD1 mice exhibit alterations in the final stages of thymocyte differentiation, as evidenced by reduced sjTREC levels in the thymus. TREC are stable, circular episomal DNA fragments formed as by-products during the rearrangement of TCR genes. These molecules are exported from the thymus to the peripheral blood as part of the recent thymic emigrants (RTE). However, since they do not replicate, their concentration decreases with successive cell divisions. Measuring TREC in peripheral blood serves as a minimally invasive method to assess thymic output, showing a strong positive correlation with the frequency of naïve CD4^+^, CD8^+^ and regulatory T cell populations (Clave et al., 2018; Douek et al., 1998; Livak and Schatz, 1996), and have been used as a marker of thymic function in the context of several immunodeficiencies and aging (Douek et al., 1998; Douek and Koup, 2000; Gaballa et al., 2016; Junge et al., 2007; Weinberg et al., 2001). In the periphery, the sjTREC/βTREC ratio has been used as a more robust indicator of the thymic output, overcoming the confounding effects that can influence sjTREC analysis alone (Dion et al., 2007; Dulude et al., 2008; Hazenberg et al., 2003). Our findings suggest that early thymocyte development is preserved and the cells progress normally through the TCRβ recombination at the DN3 stage. By contrast, the reduction in sjTREC levels indicates either impaired survival or proliferation before the DP stage, leading to fewer cells undergoing TCRα recombination (Broers et al., 2002; Dion et al., 2007). This is in accordance with our flow cytometry data showing lower numbers of DP and SP CD4^+^CD69^+^CD62L^−^ cells in the thymus. This impaired proliferation between DN and DP stages has already been reported in contexts of thymic involution, aging, and immune senescence (Clave et al., 2018; Douek et al., 1998; Junge et al., 2007). Interestingly, no apparent peripheral changes on sj/βTREC ratio were observed despite the thymic alterations, suggesting that the thymus exports comparable numbers of recent thymic emigrants, although their subset composition is probably altered, with fewer Treg, for instance (Dulude et al., 2008; Hazenberg et al., 2003). This is consistent with our observation that Treg are reduced in number in the periphery, particularly in iLN draining the hindlimbs, despite preserved Foxp3 expression and Treg/effector T cell (Teff) ratios. This result mirrors clinical findings in ALS patients, where Treg numbers are decreased at late stages, correlating with disease progression and severity (Beers et al., 2011; Henkel et al., 2013; Thonhoff et al., 2018).

**Fig. 6:**
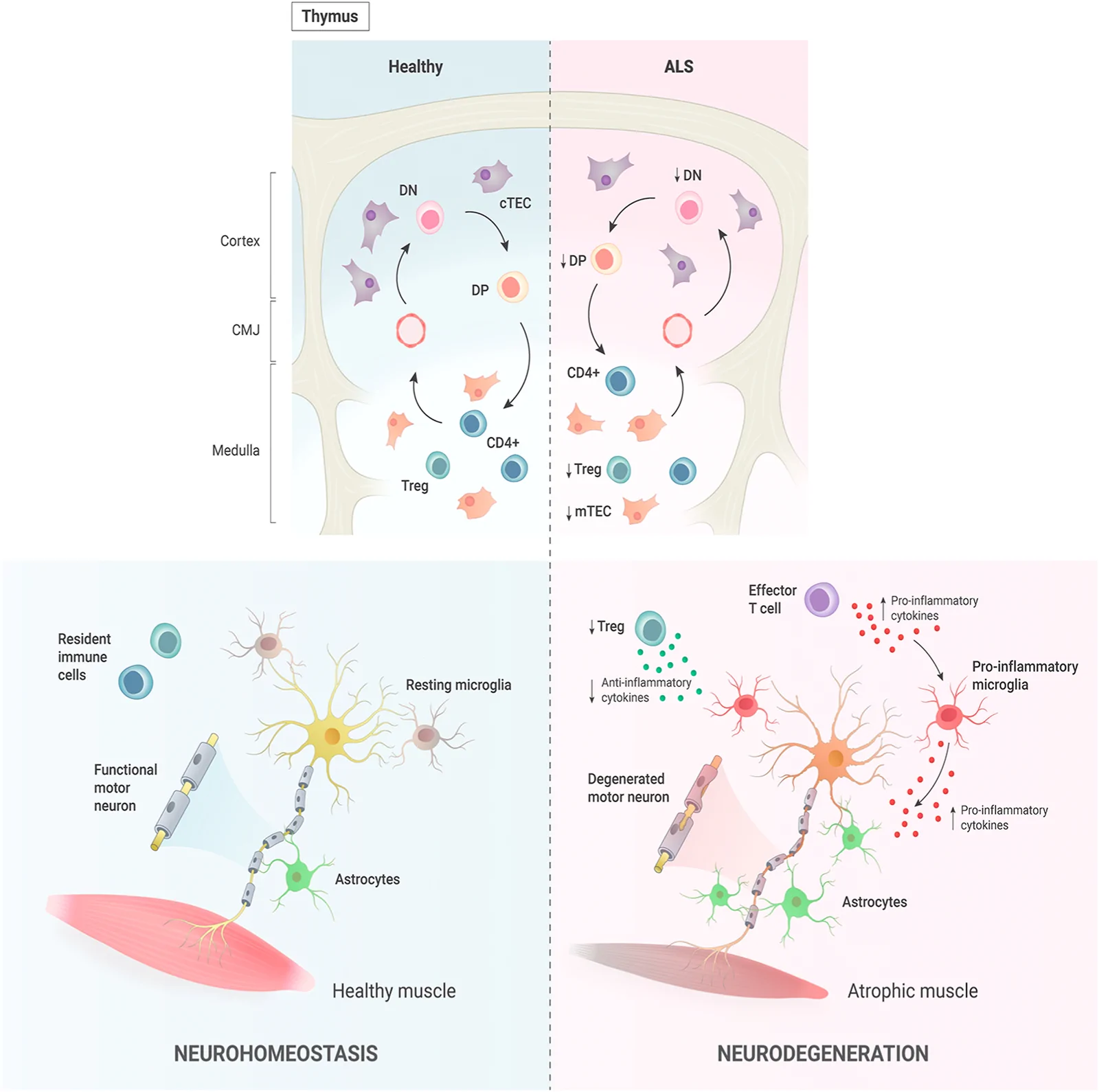
**Thymic dysfunction and immune-mediated neurodegeneration in ALS**. Schematic representation of thymic T-cell development and its impact on neuroimmune homeostasis under healthy condition *versus* ALS. In healthy (left), thymocyte maturation progresses normally within the thymus, from double-negative (DN) to double-positive (DP) stages, followed by selection into CD4^+^ T cells and regulatory T cells (Treg), supported by cortical and medullary thymic epithelial cells (cTEC and mTEC, respectively). Adequate Treg output contributes to immune tolerance and maintenance of neurohomeostasis, characterized by functional motor neurons, resting microglia, supportive astrocytes, and healthy muscle. In ALS (right), as disease progresses, thymic alterations include reduced DN and DP thymocytes, impaired thymic epithelial cell function (notably decreased mTEC), and diminished Treg generation. This imbalance favors effector T-cell responses and reduced anti-inflammatory cytokines, promoting microglial and astrocytic activation, increased pro-inflammatory cytokine release, motor neuron degeneration, and subsequent muscle atrophy.

Our results raise the possibility that ALS pathogenesis involves not only peripheral and CNS immune dysregulation but also thymic alterations and support a model where the thymus not only reflects peripheral immune changes but actively contributes to them**.** At early stages, an abundance of Treg may help restrain inflammation, consistent with reports that Treg levels are elevated in early ALS and correlate with slower disease progression (Beers et al., 2011). Since disease progresses, thymic atrophy, reduced intrathymic proliferation, TEC loss, and Treg mislocalization collectively result in reduced Treg output, which is mirrored in peripheral lymphoid tissues. This creates a cycle, in which defective central tolerance and impaired peripheral immune regulation amplify neuroinflammation, accelerating motor neuron degeneration. From a therapeutic point of view, this highlights the thymus as a possible new target for strategies aimed at restoring Treg function and rebuilding immune stability, as proposed in Fig. 6.

## 5 Conclusion

At present, ALS has no cure and treatment options remain limited. In this respect, our results highlight that the disease affects not only motor neurons but also the immune balance more broadly. Using the SOD1-G93A transgenic murine model, we demonstrate herein that ALS is associated with thymic atrophy, reduced thymocyte and Treg numbers, alterations in the thymic epithelial microenvironment and impaired Treg differentiation and suppressive function. Together, these findings suggest that thymic dysfunction may contribute to the immune dysregulation observed during disease progression and identify the thymus as a previously underappreciated component of ALS pathophysiology. Accordingly, in the future, the most effective strategies will hopefully combine established neuroprotective drugs with innovative approaches, guided by biomarkers and/or immune gene signatures.

## CRediT authorship contribution statement

**Julia Pereira Lemos:** Writing – review & editing, Writing – original draft, Visualization, Validation, Supervision, Software, Project administration, Methodology, Investigation, Formal analysis, Data curation, Writing – review & editing. **Daniel Stockholm:** Writing – review & editing, Software, Methodology, Formal analysis, Data curation. **Brenda Dinatale:** Writing – review & editing, Methodology, Formal analysis, Data curation. **Amanda Silva Chaves:** Methodology, Investigation, Data curation. **Sonia Pezet:** Resources. **Vinicius Frias Carvalho:** Writing – review & editing, Methodology, Investigation. **Ana Rosa Pérez:** Writing – review & editing, Methodology, Data curation. **Daniella Arêas Mendes-da-Cruz:** Writing – review & editing, Resources, Project administration, Funding acquisition. **Wilson Savino:** Writing – review & editing, Visualization, Validation, Supervision, Resources, Project administration, Funding acquisition, Data curation, Conceptualization. **Piera Smeriglio:** Writing – review & editing, Validation, Supervision, Funding acquisition.

## Publisher's note

All claims expressed in this article are solely those of the authors and do not necessarily represent those of their affiliated organizations, or those of the publisher, the editors and the reviewers. Any product that may be evaluated in this article, or claim that may be made by its manufacturer, is not guaranteed or endorsed by the publisher.

## Funding

This work was supported by 10.13039/501100023851Association Institut de Myologie, the Inserm-Fiocruz Associated Laboratory on Cell Therapy and Immunotherapy and the Capes-Cofecub program. WS and DAMdC are funded by 10.13039/501100003593CNPq, 10.13039/501100002322CAPES and 10.13039/501100004586FAPERJ (Brazil) and the MercoSur Fund for Structural Convergence (FOCEM). Participation of WS, DAMdC, BD, ARP, ASC and VFC is within the framework of the Brazilian National Institute of Science and Technology on Neuroimmunomodulation, the Rio de Janeiro Research Network on Neuroinflammation and the INOVA-IOC research network on Neuroimmunomodulation.

## Declaration of competing interest

The authors declare that they have no known competing financial interests or personal relationships that could have appeared to influence the work reported in this paper.

## Figures and Tables

**figs1:**
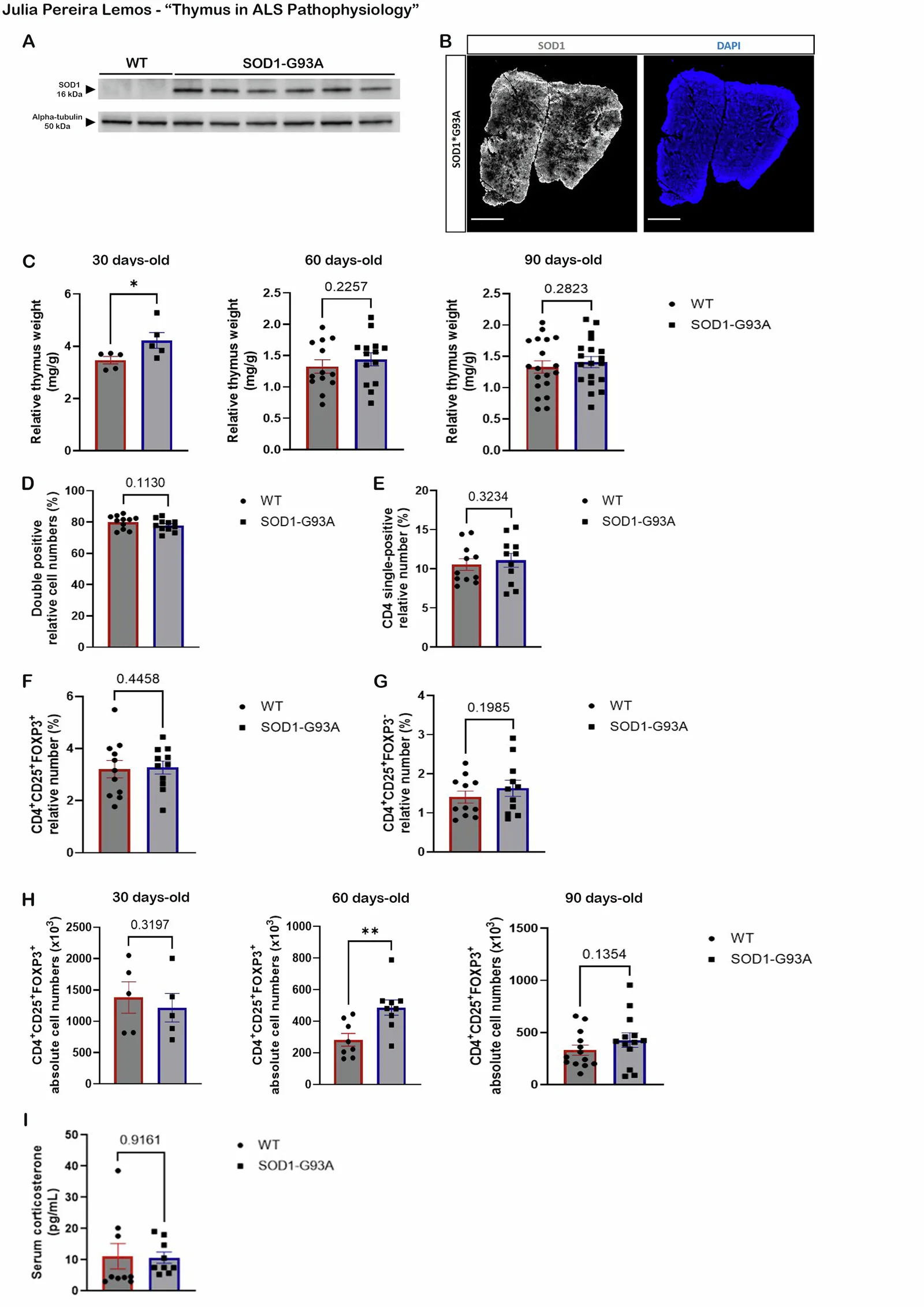
mSOD1 have higher relative thymus weight and regulatory T cell numbers in the pre-symptomatic phase of ALS. (A) Thymus protein extracts were analyzed by Western blot with human SOD1 specific antibodies. The image shows WT and SOD1-G93A representation. (B) Representative in situ hybridization of an entire thymus slice showing the mutated SOD1 expression in the SOD1-G93A mouse thymus; scale = 1 mm. (C) Relative thymus weight of B6SJLF1/J control (WT; red/ gray bar) and mSOD1 (SOD1-G93A; blue/ gray bar) male mice at 30 (left), 60 (middle) and 90 (right) days-old. (D) Relative numbers of CD4+CD8+ double-positive thymocytes in WT (red/ gray bar) and SOD1-G93A (blue/ gray bar) mice. (E) CD4 SP relative numbers in WT (red/ gray bar) and SOD1-G93A (blue/ gray bar) mouse thymi. (F-G) The graphs show regulatory T cells (CD4+CD25+Foxp3+) (F) and regulatory T cell progenitors (CD4+CD25+Foxp3-) (G) the relative numbers of in the WT (red/ gray bar) and SOD1-G93A (blue/ gray bar) mouse thymi. (H) Absolute cell numbers of regulatory T cell (CD4+CD25+Foxp3+) in the thymus of WT (red/ gray bar) and SOD1-G93A (blue/ gray bar) mice with 30 (left graph), 60 (middle graph) and 90 (right graph) days-old. (I) Quantification of corticosterone in the WT (red/ gray bar) and SOD1-G93A (blue/ gray bar) mouse sera. For western blot analyses (A), a representative image from three independent experiments is shown (WT, n = 2; mSOD1, n = 6). For in situ hybridization analyses (B), representative images from n = 4 mSOD1 mice are shown; four thymic sections per mouse were analyzed. One WT thymus was used as negative control and showed no signal (not shown). For the quantitative analyses shown in the subsequent graphs, sample sizes were: C, n = 5 WT and n = 5 mSOD1 (30 days), n = 13 WT and n = 14 mSOD1 (60 days), and n = 19 WT and n = 19 mSOD1 (90 days); D–G, n = 11 WT and n = 11 mSOD1 (120 days); H, n = 5 WT and n = 5 mSOD1 (30 days), n = 8 WT and n = 9 mSOD1 (60 days), and n = 13 WT and n = 13 mSOD1 (90 days); I, n = 9 WT and n = 9 mSOD1. Results are expressed as mean ± SEM and were analyzed by unpaired Student’s t test. Differences were considered statistically significant when ** p < 0.01.

**figs2:**
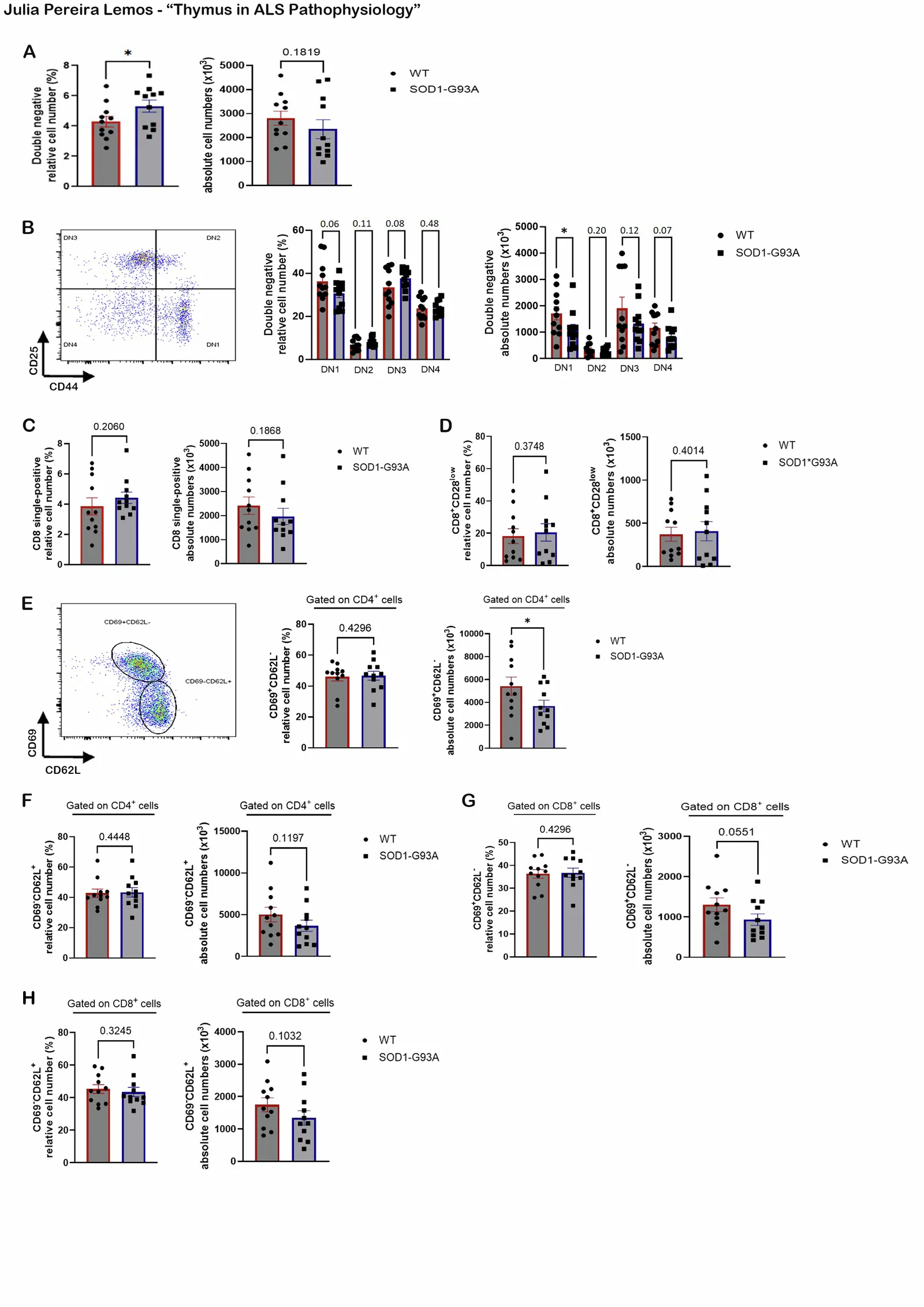
Thymocyte subpopulations in mSOD1 mice at late stage of ALS. (A) Relative (left graph) and absolute (right graph) numbers of CD4-CD8- double-negative (DN) thymocytes in WT (red/ gray bar) and SOD1-G93A (blue/ gray bar) mice. (B) Panel at left shows a representative dotplot and the gate strategy used to define the different DN subpopulations based on the CD25 and CD44 expression, analyzed by flow cytometry. The graphs show the relative (left graph) and absolute (right graph) numbers of the DN subpopulations, defined as follow: DN1 = CD25-CD44+, DN2 = CD25+CD44+, DN3 = CD25+CD44-, DN4 = CD25-CD44- in the thymus of WT (red/ gray bar) and SOD1-G93A (blue/ gray bar) mice. (C) Relative (left graph) and absolute (right graph) numbers of CD8 SP thymocytes in WT (red/ gray bar) and SOD1-G93A (blue/ gray bar) mice. (D) Relative (left graph) and absolute (right graph) numbers of thymic CD8+CD28low suppressive T cells in WT (red/ gray bar) and SOD1-G93A (blue/ gray bar) mice. (E) Panel shows a representative dotplot and the gate strategy used to define CD69+CD62L- and CD69-CD62L+ populations within CD4 and CD8 SP thymocytes, analyzed by flow cytometry. The graphs show the relative (left graph) and absolute (right graph) numbers of CD4+CD69+CD62L- cells in WT (red/ gray bar) and SOD1-G93A (blue/ gray bar) mice. (F-H) Relative (left graph) and absolute (right graph) numbers of CD4+CD69-CD62L+ (F), CD8+CD69+CD62L- (G) and CD8+CD69-CD62L+ (H) cells in the WT (red/ gray bar) and SOD1-G93A (blue/ gray bar) mouse thymi. Analyses were performed using n = 11 WT and n = 11 mSOD1 mice. Results are expressed as mean ± SEM and were analyzed by unpaired Student’s t test. Differences were considered statistically significant when * p < 0.05.

**figs3:**
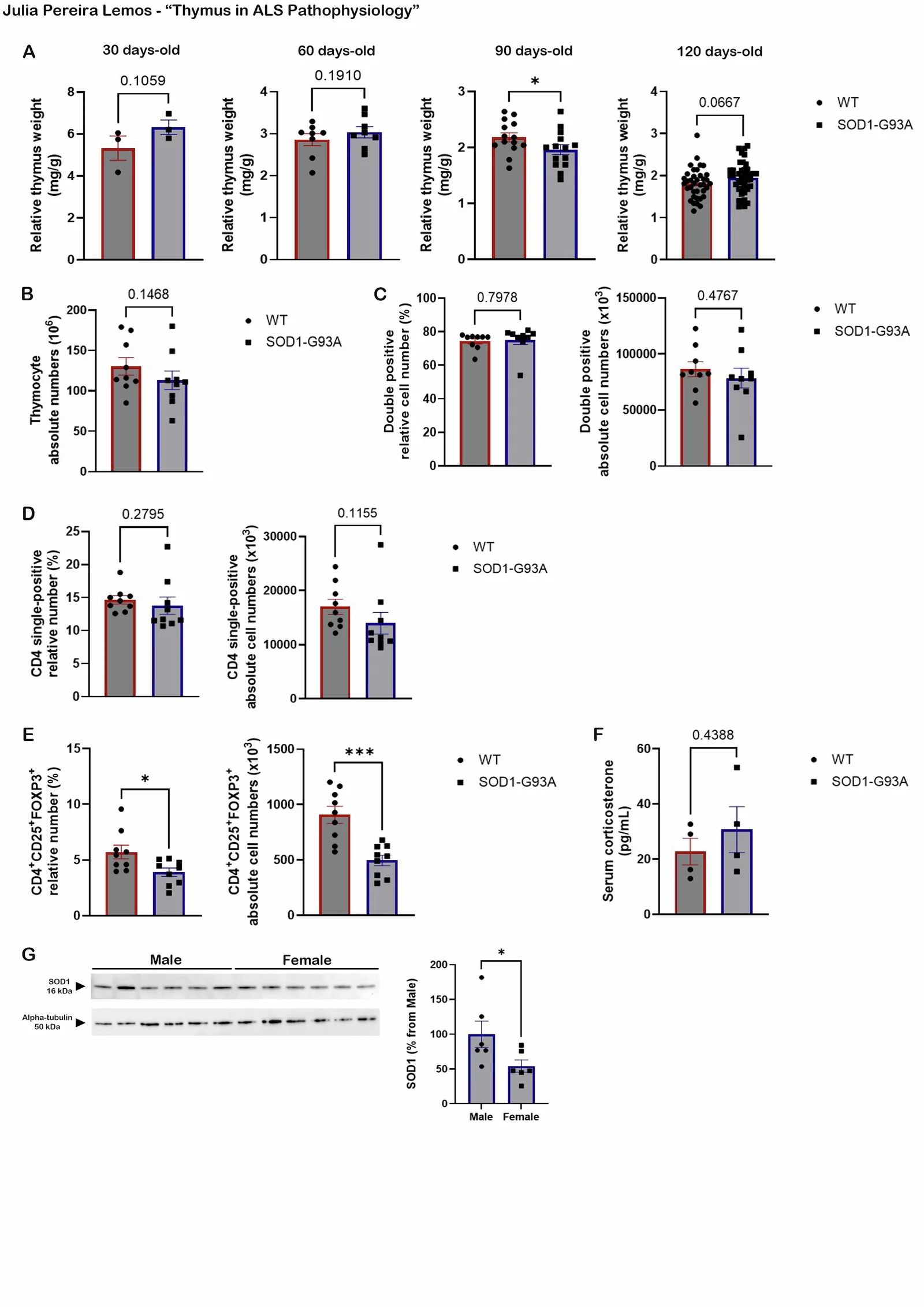
Female mSOD1 mice have different thymic implications during aging. (A) Relative thymus weight of B6SJLF1/J control (WT; red/ gray bar) and mSOD1 (SOD1-G93A; blue/ gray bar) female mice at 30 (far left), 60 (left-middle) 90 (right-middle) and 120 days-old (far right). (B) Thymocyte absolute number of WT (red/ gray bar) and SOD1-G93A (blue/ gray bar) female mice at 120 days-old. (C) Relative (left graph) and absolute (right graph) numbers of CD4+CD8+ double-positive cells in WT (red/ gray bar) and SOD1-G93A (blue/ gray bar) 120 days-old female mouse thymi. (D) Relative (left graph) and absolute (right graph) numbers of CD4 SP cells in WT (red/ gray bar) and SOD1-G93A (blue/ gray bar) female mice at 120 days-old. (E) Relative (left graph) and absolute (right graph) numbers of regulatory T cells (CD4+CD25+Foxp3+) in the thymus of WT (red/ gray bar) and SOD1-G93A (blue/ gray bar) female mice with 120 days-old. (F) Quantification of corticosterone in the WT (red/ gray bar) and SOD1-G93A (blue/ gray bar) mouse sera. (G) Male versus female thymus protein extracts were analyzed by Western blot with human SOD1 specific antibodies. The graph shows the SOD1 quantification in males and females expressed as percentage of the male expression. Sample sizes were: A, n = 3 WT and n = 3 mSOD1 (30 days), n = 8 WT and n = 9 mSOD1 (60 days), n = 14 WT and n = 14 mSOD1 (90 days), and n = 36 WT and n = 41 mSOD1 mice (120 days); B–E, n = 9 WT and n = 9 mSOD1 mice; F, n = 4 WT and n = 4 mSOD1 mice; G, n = 6 WT and n = 6 mSOD1 mice. Results are expressed as mean ± SEM and were analyzed by unpaired Student’s t test. Differences were considered statistically significant when * p < 0.05 and ***p < 0.001.

**figs4:**
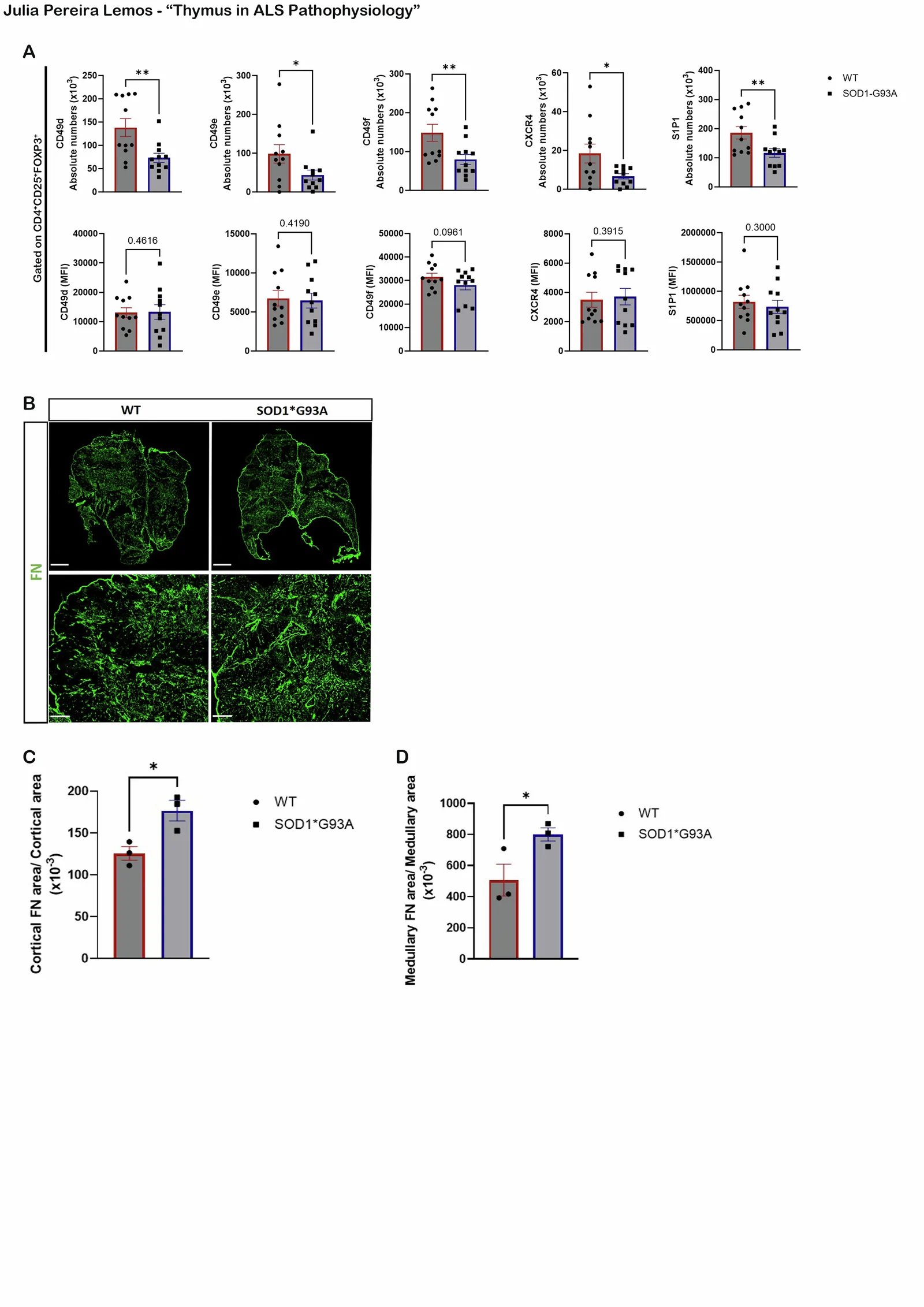
mSOD1 mouse thymus present increased fibronectin deposition at late stage of ALS. (A) The graphs show the median fluorescence intensity (MFI; top graphs) and the absolute numbers (bottom graphs) of CD4+CD25+Foxp3+ regulatory T cells expressing the CD49d, CD49e and CD49f integrins, the CXCR4 and the S1P1 receptors in the WT (red/ gray bar) and SOD1-G93A (blue/ gray bar) mice. MFI = median fluorescence intensity. (B) Representative immunohistochemistry of fibronectin (FN) expression profile in an entire slice of the thymus (top; scale = 800 μm) and in higher magnification (bottom; scale = 100 μm) showing thymic cortical and medullary regions of WT and SOD1-G93A mice. (C) Quantification of fibronectin (FN) deposition in the cortical region, normalized to total cortical area, in WT and SOD1-G93A thymus. (D) Quantification of fibronectin (FN) deposition in the medullary region, normalized to total medullary area, in WT and SOD1-G93A thymus. For flow cytometry analyses (A), n = 11 WT and n = 11 mSOD1 mice. For fibronectin immunohistochemistry analyses (B–D), n = 3 WT and n = 3 mSOD1 mice; four thymic sections per mouse, collected at 200-μm intervals, were analyzed. Results are expressed as mean ± SEM and were analyzed by unpaired Student’s t test. Differences were considered statistically significant when * p < 0.05 and ** p < 0.01.

**figs5:**
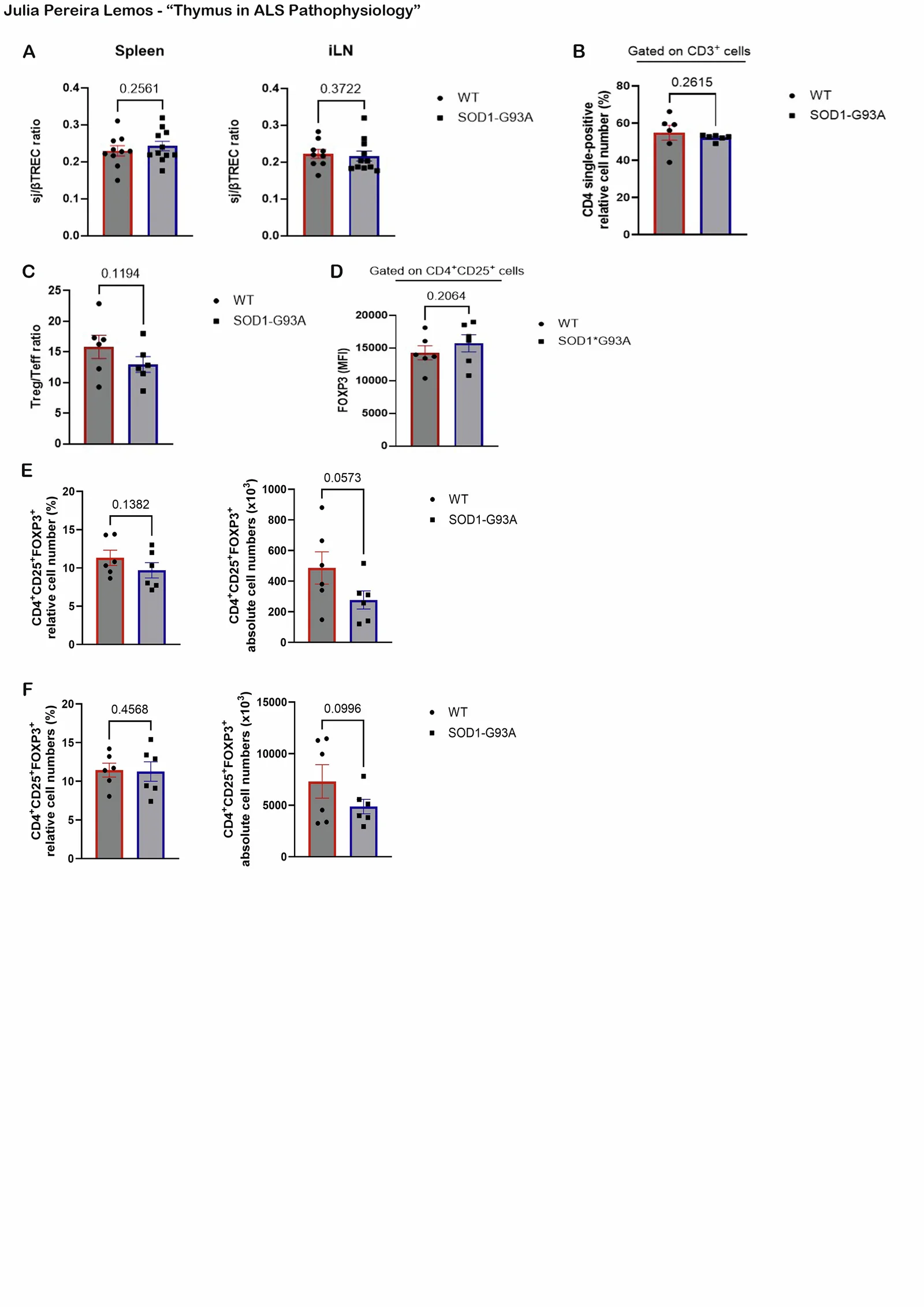
Regulatory T cell numbers in the mSOD1 peripheral organs at late stage of ALS. (A) The graphs show the sj/βTREC ratio calculated as the sum of sj61 and sj58 (sjTREC), divided by the sum of DJβ1TREC (βTREC) in the spleen (left) and inguinal lymph nodes (iLN; right) of WT (red/ gray bar) and SOD1-G93A (blue/ gray bar) mice. (B) Relative numbers of CD4 SP T cells in the WT (red/ gray bar) and SOD1-G93A (blue/ gray bar) inguinal lymph nodes (iLN). (C) Regulatory/ effector T cell ratio in the WT (red/ gray bar) and SOD1-G93A (blue/ gray bar) iLN. (D) The graph shows the Foxp3 median fluorescence intensity on CD4+CD25+ WT (red/ gray bar) and SOD1-G93A (blue/ gray bar) iLN. (E-F) Relative (left graph) and absolute (right graph) numbers of CD4+CD25+Foxp3+ regulatory T cells in the WT (red/ gray bar) and SOD1-G93A (blue/ gray bar) axillary lymph nodes (E) and spleen (F). Sample sizes were: A, n = 9 WT and n = 11 mSOD1 mice; B–F, n = 6 WT and n = 6 mSOD1 mice. Results are expressed as mean ± SEM and were analyzed by unpaired Student’s t test. MFI = median fluorescence intensity.

## Data Availability

No data was used for the research described in the article.
