# Supplementary material for: The thymus gland is a target and maintainer of amyotrophic lateral sclerosis pathophysiology
Source: Brain Behav Immun Health. 2026 Sep 6;57:101323. doi: 10.1016/j.bbih.2026.101323 (PMC13583610; doi:10.1016/j.bbih.2026.101323)
Supplement: Supplementary file 1 [file mmc1.docx]

Julia Pereira Lemos – “Thymus in ALS Pathophysiology”

**Supplementary Method 1. QuPath-Based Image Analysis, Tissue Segmentation, and Spatial Quantification**

1. **Tissue segmentation and fibronectin quantification**

To establish a ground truth dataset, small representative regions corresponding to distinct histological zones, i.e. cortex, medulla, negative biopsy and non-biopsy regions, were manually annotated. These annotations served as training data for a pixel-based random forest classification model. The trained model was subsequently applied to entire tissue sections to predict and segment these histological zones, resulting in the automated generation of region-of-interest (ROI) annotations within QuPath. To quantify fibronectin expression within each ROI, a thresholding model was applied using a manually determined threshold value of 45, selected after screening multiple representative regions, to measure the percentage area of fibronectin-positive signal, facilitating comparative analyses across the defined thymic regions.

2. **Quantification of TEC-Free, CK5⁺, CK8⁺ and Aire⁺ compartments**

To quantify the relative TEC free, CK5^+^ and CK8^+^ areas, we used specific pixel-based random forest classification models based on the following features: "Gaussian", “Laplacian", “Weighted_Std_Dev” and "Gradient_Magnitude". The models were trained to predict the area of each specific region. The quantification of the number of Aire^+^ cells was obtained by the Qupath detection method based on a threshold with the value 15 for the corresponding fluorescent channel.

3. **Foxp3⁺ cell detection and spatial analysis**

For the spatial analysis, Foxp3⁺ cells were detected using QuPath’s Cell detection plugin applied to the Foxp3 fluorescence channel with the following parameters: requested pixel size = 0.5 µm, background radius = 8 µm, median filter radius = 0 µm, sigma = 1.5 µm, minimum area = 10 µm², maximum area = 70 µm², threshold = 15, cell expansion = 5 µm. The Split by shape and Smooth boundaries options were enabled to improve segmentation accuracy. All detections were reviewed and corrected manually when necessary to remove false positives or merge over-split objects. To delineate CK5⁺ epithelial regions, a threshold-based classifier was generated using QuPath’s Create thresholder tool applied to the CK5 fluorescence channel, with Gaussian prefilter smoothing sigma = 0, threshold value = 40, “Above threshold” class assigned to CK5, “Below threshold” to background, and region set to Everywhere. The classifier was saved and applied to the images, producing annotation objects corresponding to CK5⁺ tissue areas. The centroid coordinates of Foxp3⁺ cells and the boundaries of CK5⁺ annotations were exported from QuPath. Distances from each Foxp3⁺ cell centroid to the nearest CK5⁺ annotation boundary were calculated using a custom script in R (4.4.1), ensuring Euclidean distance measurement in micrometers. Distance distributions were compared between WT and mSOD1 samples using chi^2^ test to evaluate whether Foxp3⁺ cells were positioned differently relative to CK5⁺ areas between genotypes.

To assess spatial organization, Ripley’s L-function was computed for the Foxp3⁺ cell point patterns in each sample using the spatstat package (R, version 3.2-1). The transformation L(r) – r was plotted as a function of the spatial scale r to visualize clustering or dispersion compared to complete spatial randomness (CSR). Differences in clustering between genotypes were evaluated by comparing the mean L(r) – r curves and their confidence envelopes derived from Monte Carlo simulations (n = 10).
