# Supplementary material for: The thymus gland is a target and maintainer of amyotrophic lateral sclerosis pathophysiology
Source: Brain Behav Immun Health. 2026 Sep 6;57:101323. doi: 10.1016/j.bbih.2026.101323 (PMC13583610; doi:10.1016/j.bbih.2026.101323)
Supplement: Supplementary file 2 [file mmc2.docx]

Julia Pereira Lemos – “Thymus in ALS Pathophysiology”

**Supplementary Method 2. Linear Discriminant Analysis (LDA) Models and LD1 Score Calculation**

For reproducibility, the final LD1 equations derived from the linear discriminant analysis (LDA) models are provided below. The equations are expressed in the original measurement units using raw coefficients and intercepts, allowing direct calculation of LD1 scores from new datasets without re-scaling.

1. **Thymocyte Dataset**

Variables included: total thymocytes, CD4, CD8, DP, DN (DN1–DN4), Foxp3 MFI, Treg, Treg progenitors, CD69 versus CD62L subsets and ROW.

$$\begin{matrix} LD1= & -14.45+2.71\cdot\text{row120d}-0.26\cdot\text{Thymocytes} \\ & -0.036\cdot\text{TregProg}+0.0048\cdot\text{Treg120d}+0.0034\cdot\text{DN1} \\ & -0.0074\cdot\text{DN2}+0.0009\cdot\text{DN3}-0.0006\cdot\text{DN4} \\ & +0.0010\cdot\text{DN}+0.0029\cdot\text{CD69CD62L\_CD8}-0.0016\cdot\text{CD69CD62L\_CD4} \\ & +0.0016\cdot\text{CD8}+0.0001\cdot\text{CD4}+0.0004\cdot\text{DP}+0.0009\cdot\text{Foxp3\_MFI} \end{matrix}$$

2. **Epithelial Dataset**

Variables included: ROW, Total cell count, EpCAM, cTEC, mTEC, mature mTEC, MFI_Aire_Mature.mTEC.

$$\begin{matrix} LD1= & -9.92-0.41\cdot\text{ROW}+0.19\cdot\text{EpCAM}-0.20\cdot\text{cTEC}-0.20\cdot\text{mTEC} \\ & +0.11\cdot\text{Cell.count}+0.028\cdot\text{Mature.mTEC}+0.00041\cdot\text{MFI\_Aire\_Mature.mTEC} \end{matrix}$$

In both datasets, LD1 provides a synthetic score that maximally separates genotypes: higher LD1 values correspond to WT, while lower values correspond to SOD1-G93A. These scores were used for visualization and statistical comparison in Figures 1I and 2L.
