# Supplementary material for: The thymus gland is a target and maintainer of amyotrophic lateral sclerosis pathophysiology
Source: Brain Behav Immun Health. 2026 Sep 6;57:101323. doi: 10.1016/j.bbih.2026.101323 (PMC13583610; doi:10.1016/j.bbih.2026.101323)
Supplement: Supplementary file 3 [file mmc3.docx]

Julia Pereira Lemos – “Thymus in ALS Pathophysiology”

| **Supplementary Table 1: Antibodies and molecules** | | | |
| --- | --- | --- | --- |
| **Flow cytometry/ Cell sorting** | | | |
| **Antibody/ Molecule** | **Clone** | **Supplier** | **Catalog number** |
| Live/Dead Zombie Aqua | - | Biolegend | 423102 |
| Live/Dead Zombie Yellow | - | Biolegend | 423104 |
| AIRE AF647 | MM-525 | BD Biosciences | 567253 |
| CD16/CD32 (Fc Block) | 2.4G2 | BD Biosciences | 553142 |
| CD19 PE-CF594 | 1D3 | BD Biosciences | 562291 |
| CD25 APC | PC61.5 | Invitrogen | 17-0251-82 |
| CD28 purified | 37.51 | BD Biosciences | 557393 |
| CD3 PE-Cy7 | 500A2 | Biolegend | 152314 |
| CD4 BV785 | GK1.5 | Biolegend | 100453 |
| CD4 Pacific Blue | RM4-5 | Biolegend | 100531 |
| CD4 PE | RM4-5 | Biolegend | 100512 |
| CD44 APC-R700 | IM7 | BD Biosciences | 565480 |
| CD45 FITC | 30-F11 | Biolegend | 103108 |
| CD45 PerCP-Cy5.5 | 30-F11 | Biolegend | 103132 |
| CD45RB PE | C363-16A | Biolegend | 103308 |
| CD62L APC-Cy7 | MEL-14 | BD Biosciences | 560514 |
| CD69 BV421 | H1.2F3 | Biolegend | 104528 |
| CD8 AF700 | 53-6.7 | Biolegend | 100730 |
| CD8 BV510 | 53-6.7 | Biolegend | 100752 |
| CD80 PE | 16-10A1 | Biolegend | 104708 |
| CXCR4 CF-CF594 | 2B11/CXCR4 | BD Biosciences | 565019 |
| EpCAM APC-Cy7 | G8.8 | Biolegend | 118218 |
| FOXP3 PE | FJK-16s | Invitrogen | 12-5773-82 |
| Ly-51 PE-Cy7 | 6C3 | Biolegend | 108314 |
| MHCII BV421 | M5/114.15.2 | Biolegend | 107632 |
| S1P1 purified | Polyclonal | Invitrogen | PA1-1040 |
| TER119 AF700 | TER-119 | Biolegend | 116220 |
| UEAI FITC | Polyclonal | Vector Laboratories | FL-1061 |
| VLA-4 PE-CF594 | R1-2 | BD Biosciences | 564395 |
| VLA-5 PE-CF594 | 5H10-27 (MFR5) | BD Biosciences | 564313 |
| VLA-6 PE-Cy5 | GoH3 | BD Biosciences | 551129 |
| Goat anti-rabbit AF488 | Polyclonal | Invitrogen | A-11034 |
| Goat anti-hamster AF488 | Polyclonal | Abcam | ab173003 |
| **Immunofluorescence *in situ*** | | | |
| **Antibody** | **Clone** | **Supplier** | **Catalog number** |
| AIRE AF488 | 5H12 | Invitrogen | 53-5934-82 |
| CK5 AF647 | EP1601Y | Abcam | ab193895 |
| CK8 AF488 | EP1628Y | Abcam | ab192467 |
| Fibronectin | Polyclonal | Sigma-Aldrich | F3648 |
| FOXP3 | FJK-16s | Invitrogen | 14-5773-82 |
| Laminin | Polyclonal | DAKO | Z0097 |
| Goat anti-rabbit AF488 | Polyclonal | Invitrogen | A-11034 |
| Donkey anti-rat AF488 | Polyclonal | Invitrogen | A-21208 |
| **Western Blotting** | | | |
| **Antibody** | **Clone** | **Supplier** | **Catalog number** |
| Alpha-tubulin | B-5-1-2 | Sigma-Aldrich | T5168 |
| Human SOD1 | G215-1 | BD Biosciences | 556360 |
| Sheep anti-mouse HRP | Polyclonal | VWR | NA931 |
| ***In vitro* differentiation assay** | | | |
| **Antibody/ Molecule** | **Clone** | **Supplier** | **Catalog number** |
| Ultra-LEAF CD28 | 37.51 | Biolegend | 100372 |
| Ultra-LEAF CD3 | 145-2C11 | Biolegend | 102132 |
| Recombinant Mouse IL-2 | - | R&D Systems | 402-ML |
| Recombinant Mouse TGF‑β1 | - | R&D Systems | 7666-MB |
