# Supplementary material for: The thymus gland is a target and maintainer of amyotrophic lateral sclerosis pathophysiology
Source: Brain Behav Immun Health. 2026 Sep 6;57:101323. doi: 10.1016/j.bbih.2026.101323 (PMC13583610; doi:10.1016/j.bbih.2026.101323)
Supplement: Supplementary file 4 [file mmc4.docx]

Julia Pereira Lemos – “Thymus in ALS Pathophysiology”

| **Supplementary Table 2: Primers applied for PCR analyses** | |
| --- | --- |
| **Gene** | **Sequence** |
| CD4 Outer 1 | CCAACCAACAAGAGCTCAAGGA |
| CD4 Inner 1 | AGCTCAAGGAGACCACCATGT |
| CD4 Outer 2 | CCCAGAATCTTCCTCTGGT |
| CD4 Inner 2 | TGGTCAGAGAACTTCCAGGT |
| δREC Outer | AGTGTGTCCTCAGCCTTGAT |
| δREC Inner | GAAAACCTCCCCTAGGAAGA |
| sjTREC Jα58 Outer | CCCAGGACACCTAAAAGGAT |
| sjTREC Jα58 Inner | AACTCGCACAGTGGAGGAAA |
| sjTREC Jα61 Outer | AACTGCCTGGTGTGATAAGAT |
| sjTREC Jα61 Inner | GGAGTATCTCTTTGGAGTGA |
| Dβ1 Outer | TATCCACTGATGGTGGTCTGTT |
| Dβ1 Inner | GACGTTGGCAGAAGAGGATT |
| Jβ 1.1 Outer | CATGTTTGACATTGCCACAAGT |
| Jβ 1.1 Inner | AGCGATTACTCCTCCTATGGT |
| Jβ 1.2 Outer | CTCTCTTCACCCCTTAAGATT |
| Jβ 1.2 Inner | GGTAAAGGAACCAGACTCACAGTT |
| Jβ 1.3 Outer | TGAGGCTGGATCCACAAAGGT |
| Jβ 1.3 Inner | TCAAGATGAACCTCGGGTGGA |
| Jβ 1.4 Outer | GGGCCATTAGGAAACGTGAT |
| Jβ 1.4 Inner | GCAGGAAGCATGAGGAAGTT |
| Jβ 1.5 Outer | GGAGGAAGGAAGGATGGTGA |
| Jβ 1.5 Inner | CAGAGTCCTGCCTCAAAGAA |
| Jβ 1.6 Outer | CCTGTGACATGCCTCATGGTA |
| Jβ 1.6 Inner | TCAGGTCTCAGGGATCTAAGA |
